# Retrograde rearrangement of mitochondria correlates with nuclear deformation and genotoxic damage

**DOI:** 10.1016/j.isci.2025.112955

**Published:** 2025-06-19

**Authors:** Maximilian Jobst, Francesco Crudo, Doris Marko, Andrea Bileck, Samuel Matthias Meier-Menches, Christopher Gerner, Giorgia Del Favero

**Affiliations:** 1 University of Vienna, Faculty of Chemistry, Department of Food Chemistry and Toxicology, Währinger Str. 38, 1090 Vienna, Austria; 2 University of Vienna, Vienna Doctoral School in Chemistry (DoSChem), Währinger Str. 42, 1090 Vienna, Austria; 3 University of Vienna, Faculty of Chemistry, Core Facility Multimodal Imaging, Josef-Holaubek-Platz 2, 1090 Vienna, Austria; 4 University of Vienna, Faculty of Chemistry, Department of Analytical Chemistry, Währinger Str. 38, 1090 Vienna, Austria; 5 Joint Metabolome Facility, University of Vienna and Medical University Vienna, Währinger Str. 38, 1090 Vienna, Austria; 6 University of Vienna, Faculty of Chemistry, Department of Inorganic Chemistry, Währinger Str. 38, 1090 Vienna, Austria

**Keywords:** Biological sciences, Biochemistry, Cell biology, Toxicology, Biophysics

## Abstract

The elucidation of molecular mechanisms underlying DNA damage and repair belongs to the fundamental questions of pathophysiology and toxicology. Increasing attention is focusing on the role of mechanical stress exerted on the nucleus and its function as a mechanosensor. Hypothesizing that physical cues arising from the intracellular rearrangements could contribute to the genotoxic damage, we observed that the retrograde relocalization of the mitochondria coincides with increased nuclear stiffness in T24 bladder cells. Perinuclear mitochondrial clustering aligned with the deformation of the nucleus and was accompanied by DNA strand breaks. In the tested experimental layouts, these events appeared scarcely dependent on oxidative stress, strengthening a possible contribution of mechanical nuclear deformation. Proof of principle experiments in SK-OV-3 and HCT 116 cells underpinned the role of cellular architecture and its heterogeneity. These findings open new avenues for understanding how physical changes in the intracellular compartment may drive genotoxicity, potentially supporting genetic instability and carcinogenesis.

## Highlights

• Bafilomycin induces a retrograde rearrangement of the mitochondrial network
• Mitochondrial mass shifts relate to changes in nuclear morphology and stiffness
• Nucleoskeletal deformation is accompanied by DNA damage
• Genotoxicity and morphometric adaption in ovarian and bladder cancer cells

## Graphical abstract

**Figure:**
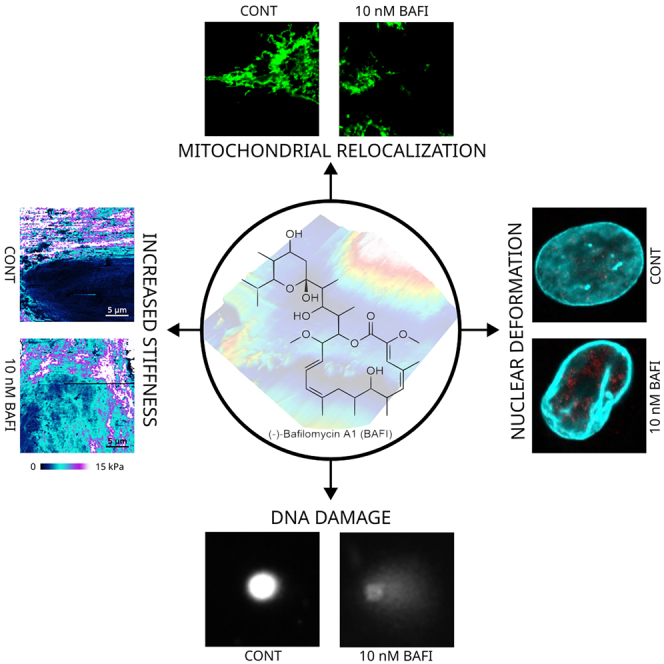

## Introduction

The evaluation of the genotoxic potential is one of the fundamental steps for the classification of compounds by governmental institutions (e.g., EURL ECVAM,^1^ OECD,^2^ and EPA^3^) and one of the most widely used endpoints to assess the toxicity of chemicals. Aiming at detecting a DNA lesion, genotoxicity testing^4^^,^^5^ has been routinely used in the field of chemical carcinogenesis for decades and has supported the identification and understanding of human carcinogens aligning experimental findings to epidemiological data. However, until now, the field largely focused on pathways related to chemical reactions between DNA and endogenous or exogenous molecules and the following formation of DNA adducts, base deletions, or strand breaks.^6^

In addition to chemical triggers, a possible role for physically induced DNA damage is starting to emerge.^7^^,^^8^^,^^9^^,^^10^ This expands the existing knowledge on ionizing radiations toward biophysical considerations related to cell structure and integrity.^11^ The latter is of great physiological relevance as cells are constantly exposed to mechanical cues *in vivo*, for instance in relation to blood or lymphatic flow, pressure, and deformation during tissue regeneration, senescence, or adjustment of organs within the body.^12^^,^^13^^,^^14^^,^^15^^,^^16^ Stemming from mechanotransduction-related pathways, namely from the conversion of physical cues into biochemical signaling, it is possible to regulate apoptosis, tissue repair, migration, epithelial–mesenchymal transition (EMT), to name some examples.^17^^,^^18^^,^^19^^,^^20^ This includes the contribution of several key molecular effectors and among these, the nucleus has a central role in supporting cell mechano-sensation.^21^ The nucleus is surrounded by the nuclear envelope, supported by A-type and B-type Lamins. Diseases connected to mutations of these proteins manifest phenotypically in the loss of function of mechanically active tissues, as for example with the loss of mechanical stability in cardiomyocytes and the onset of cardiomyopathies.^22^^,^^23^^,^^24^ This correlation provided essential contributions for understanding the role of the nucleus in mechanotransduction.^25^ Fittingly, Lamin A/C deficient cells (Lmna −/−) display increased nuclear deformation, defective mechanotransduction, and reduced viability under mechanical strain.^26^ However, mechanotransduction can be hardly described by refraining to single entities: there is a general consensus in describing response to physical cues rather as a chain of events connecting extracellular to intracellular compartments.^27^^,^^28^ Following this, physical signals from the cell membrane can be passed on from intermembrane proteins (i.e., integrins^29^), which stand in direct contact with the extracellular matrix, toward connections between the cytoskeleton and the nucleus, through the linker of nucleoskeleton and cytoskeleton complex (LINC).^30^^,^^31^ The cytoskeletal filaments do not only act as force transducers, but also help to stabilize the nuclear structure against compression.^32^

These adaptation mechanisms are necessary since cell strain and compression can occur in pathophysiological settings *in vivo*. In cell collectives, as those composing tightly packed tumors, cells are relatively rigid and immobile. For cancer cell migration to be possible, a change of the physical properties must take place, and the tissue undergoes a process called “unjamming”.^33^ In this case, a solid-to-liquid transition starts, allowing the cells to move freely and supporting the interstitial dissemination of cancer cells.^33^ Even without considering the contributions of fluid shear stress or ECM stiffness, the unjamming process alone is a source of mechanical stress and nuclear deformation.^34^ Likewise, single cells migrating through tightly packed tissues experience similar mechanical stress.^7^^,^^35^ The increased pressure on the nucleus during these processes can have significant effects on the nuclear structure and stability. Firstly, short term nuclear envelope (NE) ruptures can occur, exposing the chromatin to the contents of the cytoplasm.^7^^,^^34^ Contact with cytoplasmic factors can result in DNA damage to the unprotected genetic material. However, the leak is generally rapidly repaired through Endosomal Sorting Complex Required for Transport III (ESCRT III).^36^ The effective repair mechanism might be key to the high survivability displayed by cancer cells despite NE rupture.^36^ In this view, a weakened NE, as indicated by reduced Lamin A/C expression, might even present an advantage for migrating cancer cells as a more deformable nucleus allows easier passage through confined spaces during metastasis, while the increased genomic instability may further foster the onset of mutations and aggressive phenotypes.^37^ Additionally, the compression itself can also interfere with nuclear dynamics. Recent lines of evidence indicate that nuclear deformation on its own is sufficient to cause DNA damage.^7^^,^^8^ Increased replication stress and stalled replication forks are the suspected causes in this case. In more detail, the increased external pressure may change DNA conformation, increase DNA torsional stress and cause the stalling or collapse of the replication forks.^8^

Considering tissues characterized by high mechanical load, cells in the bladder are exposed to multiple stimuli, including fluid shear stress from the urine and deformation cycles of the tissue during filling and voiding of the organ.^38^ In this light, bladder cancer is also defined by the biomechanics of the surrounding environment. For instance, tissue deformation stemming from mechanical instability at the onset of bladder tumor development can be another source of physical cues.^39^ These mechanical stressors coexist with the high chemical variability of the urine composition, including low nutrient availability and the presence of bioactive metabolites and contaminants.^40^^,^^41^ Bladder cells extensively rely on sophisticated adaptive responses, making them especially suited for the investigation of intertwined chemical and physical signaling pathways.^42^^,^^43^

Deepening on molecular effectors regulating plasticity of bladder cells, the nucleoskeleton integrity and lamin function are especially relevant; a reduction in Lamin A expression can induce bladder muscle dystrophy^44^ and a role for Lamin A is being discussed in bladder cancer.^45^^,^^46^ In this regard, Lamin A expression is reduced in non-invasive bladder tumor tissues, and Lamin A deficiency can promote the muscle invasion of bladder cancer cells.^45^^,^^46^

Hunting key molecular events potentially connecting nuclear mechanical stability to DNA integrity, notably less attention has been devoted so far to the potential role of rearrangements of intracellular compartments. In bladder cells, shifts in the area occupied by the endoplasmic reticulum (ER)^43^ or the mitochondrial network^42^ may occur in relation to several chemical exposures.

The ER has been described as an important integration site of mechanotransduction, its distribution throughout the cytosol allows for significant deformation, and its close interconnectedness with the cytoskeleton and the nucleus infers for the transfer of mechanical forces.^47^^,^^48^ Furthermore, the ER possesses multiple mechanosensitive ion channels in its membrane, including PIEZO1^49^^,^^50^ and Pannexin-1.^51^ Additionally, there is evidence that the ER can contribute to the physical properties of cells directly; in this respect, it was previously described that rearrangements of the ER toward cell nucleus or periphery are accompanied by subcellular changes in cells stiffness: for example, the application of thapsigargin (2 h, 100 nM) increases the Young’s modulus in the nuclear and perinuclear areas corresponding to the accumulation of ER and actin displacement.^52^ Adhering to the idea that more general mechanisms could exist connecting non-cytoskeletal organelles with mechanotransduction,^53^ a contribution was envisioned for the mitochondrial network. The biophysical properties of the organelles can alter in respect to fission and fusion dynamics,^54^ movement of mitochondria^55^^,^^56^^,^^57^ or by the alteration of individual mitochondria’s physical properties.^58^^,^^59^ Furthermore, mitochondria are closely interconnected with the ER, the nucleus and the cytoskeleton which makes the transfer of forces likely.^60^^,^^61^^,^^62^^,^^63^^,^^64^ Endowing a bi-directional relationship, physical cues can trigger changes in mitochondrial dynamics or function and mitochondrial mechanotransduction can play a role in the cellular response to physical stimuli,^64^^,^^65^^,^^66^^,^^67^ yet, the contribution of the organelles themselves as a source of physical cues remains largely unrevealed.

In the T24 bladder cancer cell line, tailored mitochondrial rearrangement was achieved with the V-ATPase and autophagy inhibitor bafilomycin A1.^68^ Building on this, the study at hand explores the contribution of mass relocation triggered by mitochondrial retrograde movement toward the nuclear compartment and the coherent adaptation of the nuclear morphology as well as the occurrence of DNA damage. Untargeted proteome analysis of bladder nuclear extracts described the accumulation of mitochondrial proteins together with a signature of altered cell division, DNA replication, DNA repair, and DNA damage. This aligned with the alteration of cell stiffness, nuclear and Lamin A/C morphometric adaptations, and the presence of DNA damage.

## Results

### Effects of bafilomycin on mitochondrial network reorganization and nuclear proteome in T24 bladder cancer cells

**Figure 1:**
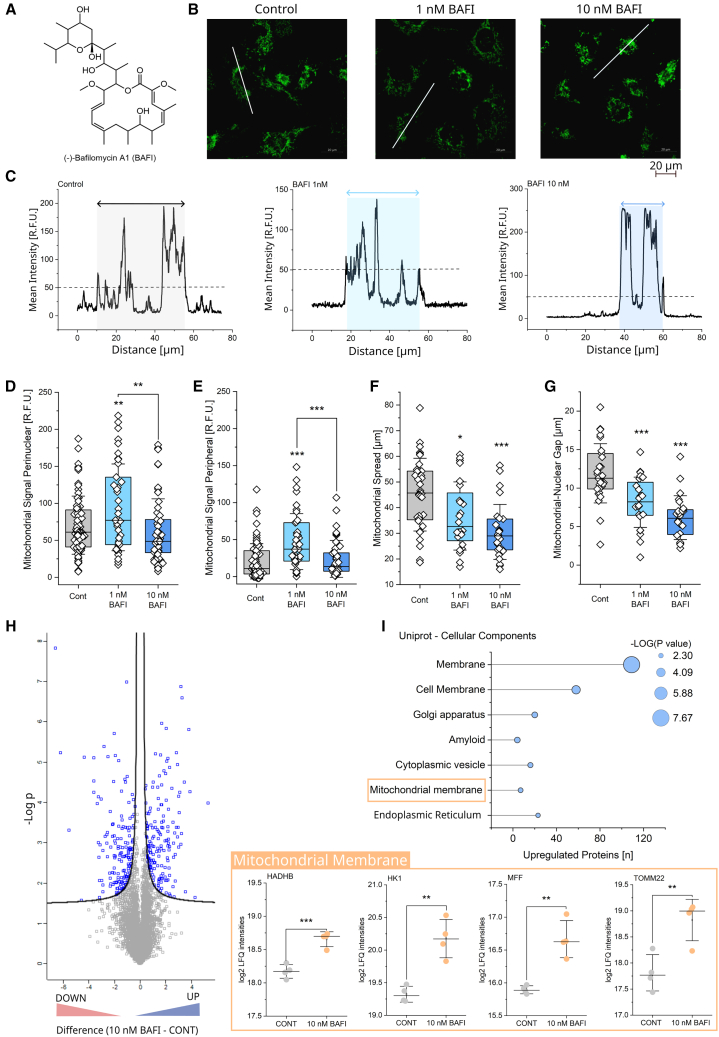
Mitochondrial network reorganization in T24 bladder cancer cells following 24 h bafilomycin treatment (A) Structure of bafilomycin A1 (PubChem CID 6436223). (B) Representative images of MitoTracker stained mitochondria (green) in T24 control cells and cells treated with bafilomycin (1–10 nM) for 24 h. Lines indicate representative cross-sections along the major axis of the cell selected for the quantification in Figures 1C, 1F, and 1G. Scale bar 20 μm. (C) Graphical representation of the intensity profiles across of selected cross-sections, showing the signal intensity (R.F.U.) over the distance (μm); highlighted is the measurement of the mitochondrial spread (dotted line marks reference threshold of 50 R.F.U.). (D) Quantification of the signal intensity (R.F.U.) in the perinuclear area of the cells. (E) Quantification of the signal intensity in the cell periphery. *N* ≥ 38 ROIs (Figures 1D and 1E) per condition were evaluated, and at least 3 biological replicates were performed. (F) Quantification of the maximum spread (μm) of the network. (G) Quantification of the nuclear gap (μm) inside the mitochondrial signal. For the gap and spread of the network *n* > 25 cells were quantified per condition (Figures 1F and 1G). Data were taken from at least three independent biological replicates. Results (Figures 1D–1G) are shown as boxplots, whiskers represent SD, and boxes represent the range from 25 to 75 percentage. Statistical significance was determined using a one-way ANOVA test with Fisher’s LSD test for means comparison (∗*p* < 0.05, ∗∗*p* < 0.01, ∗∗∗*p* < 0.001). (H) Volcano Plot of the proteome analysis of nuclear extracts of controls compared to bafilomycin treated (10 nM) cells (24 h), individual dots represent down (left side) or up (right side) regulated proteins. Y axis represents -Log(P) while x axis describes the difference between protein levels (log2). Blue dots above the border line represent proteins that are significantly regulated, gray not significantly regulated proteins. (I) DAVID analysis of the upregulated proteins, for the uniport keywords of the category cellular components. On the x axis, the number of regulated proteins, the size of the symbols indicates the statistical significance (-Log(P)). Highlighted is the upregulation of proteins of the mitochondrial membrane. Additional results of the DAVID analyses (Uniprot keywords: molecular function and PTM) can be found in Figure S1. Label-free quantification (LFQ) values of selected proteins from the category “mitochondrial membrane”, i.e., HK1, HADHB, MFF, and TOM22. Whiskers represent SD, statistical significance was determined via Student’s *t*-Test (∗*p* < 0.05, ∗∗*p* < 0.01, ∗∗∗*p* < 0.001). Samples for MS analysis were prepared in quadruplicate.

In T24 bladder cancer cells, the mitochondrial network is sensitive to autophagy modulation.^40^^,^^42^ After applying previously established non-cytotoxic concentrations (1–10 nM, 24 h)^40^ of the autophagy inhibitor bafilomycin A1 (BAFI, Figure 1A), a morphometric rearrangement of the mitochondria was obtained in living cells observable with MitoTracker using confocal microscopy (Figures 1B and 1C). Evaluation of the mitochondrial signal intensities in selected subcellular regions of interest (ROIs) revealed that even in control conditions, the signal in the perinuclear ROIs was higher than the one in the cell’s periphery. Upon incubation with 1 nM bafilomycin, an increase in the signal was detected in both compartments (Figures 1D and 1E). Focusing on the intracellular shift in the signal intensity, the morphology of the network was investigated. To assess the overall spread of the network within the cells (Figure 1F), the maximum diameter of the mitochondrial signal footprint was determined along the major axis of the cells. Both tested concentrations of bafilomycin (1–10 nM) reduced the diameter of the network in a concentration dependent fashion (Figures 1C and 1F). Additionally, to explore the rearrangement in the nuclear proximity, the gap in the mitochondrial signal distribution due to the presence of the nucleus was quantified. Coherently, this parameter decreased significantly upon bafilomycin incubation (Figure 1G). Overall, these two parameters together indicate a rearrangement of the network from the cell periphery toward the nuclear/perinuclear area. To confirm the mitochondrial relocation and start investigating the cellular downstream effects stemming thereof, an untargeted proteome profiling of nuclear extracts was performed (Figure 1H). The analysis enabled the identification of in total 4898 proteins and 24 h incubation with 10 nM bafilomycin returned multiple regulatory events (Figure 1H; 161 up- and 162 downregulated proteins). In order to identify cell functions and biological processes that can be traced back to the proteome signature a Database for Annotation, Visualization and Integrated Discovery (DAVID)^69^^,^^70^ bioinformatics analysis was performed (Figure 1I). Supportive of an extensive intracellular rearrangement toward the nuclear region, the treatment with bafilomycin returned a significant enrichment of several cellular components, including membranes, the Golgi apparatus, and constituents of the endoplasmic reticulum (Figure 1I). Importantly for our analysis, the proteome data confirmed an increase in mitochondrial membrane proteins in the nuclear fraction of the cell lysate (Figure 1I), aligning with the repositioning of the mitochondrial network detected by live cell imaging. Examples of proteins with an elevated presence in the nuclear fraction included; the Hexokinase 1 (HK1), primarily found attached to mitochondrial membranes,^71^ the HADHB a subunit of the mitochondrial trifunctional protein,^72^ as well as the mitochondrial fission protein (MFF)^73^ and the mitochondrial import receptor subunit TOM22 homolog (hTom22)^74^ (Figure 1I).

### Comparison between bafilomycin and H_2_O_2_ induced nuclear proteome signatures

**Figure 2:**
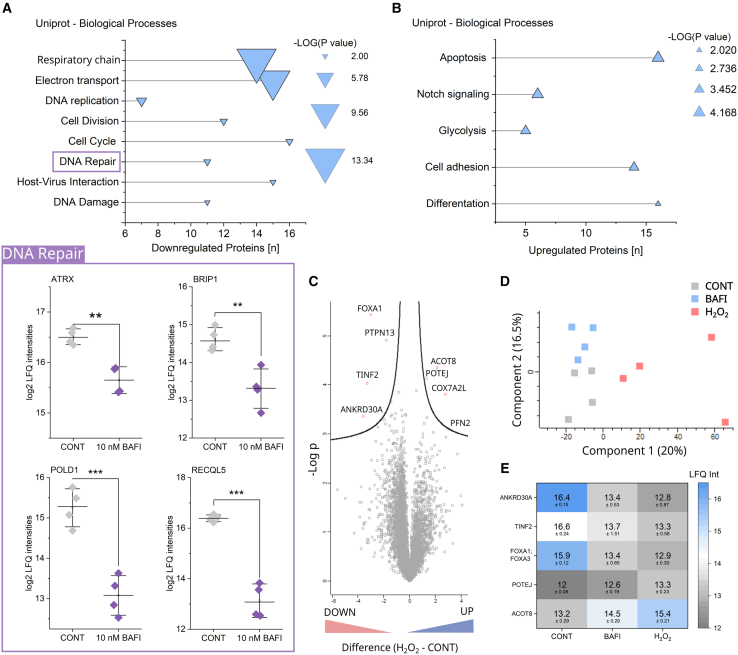
Proteome profile of T24 cells treated with bafilomycin for 24 h (A) DAVID analysis of regulated proteins in cells incubated with bafilomycin (10 nM for 24 h) vs. control cells. Downregulated protein categories according to the Uniprot keywords “Cellular processes.” On the x axis, the number of regulated proteins, the size of the symbols indicates the statistical significance (-Log(P)). Selected downregulated proteins of the category DNA repair, i.e., ATRX, BRIP1, POLD1, and RECQL5. Whiskers depict SD, statistical significance was determined via Student’s *t*-Test (∗∗*p* < 0.01, ∗∗∗*p* < 0.001). (B) Upregulated protein categories according to the DAVID analysis using the Uniprot keywords “Cellular processes”. (C) Volcano plot of the proteome analysis of nuclear extracts of controls compared to H_2_O_2_ treated (0.5 mM) cells, individual dots represent down- (left side) or upregulated (right side) proteins. Y axis represents -Log(P), while x axis describes the difference between protein levels (log2). Blue dots above the border line represent proteins that are significantly regulated, while not significantly regulated proteins are depicted in gray. (D) Principal component analysis (PCA) of control cells and cells treated with bafilomycin (10 nM) or H_2_O_2_ (0.5 mM) for 24 h. (E) Heatmap listing mean LFQ values and standard deviations of the proteins regulated in both bafilomycin and H_2_O_2_ treated cells, compared to control cells (Figures 2A–2E). Cell preparations for MS analysis were prepared in quadruplicate.

Deepening on the significantly regulated proteins in T24 cells incubated with bafilomycin, further analysis revealed a decrease in respiratory and electron transport chain proteins, supporting the dysfunction of the mitochondria and possibly a connection to the reorganization of the network (Figure 2A). Furthermore, a signature suggestive of replication stress could be observed, i.e., decreased expression in the proteins of the categories DNA replication, cell division, and cell cycle. Changes in the biological processes associated to DNA repair and DNA damage indicate potential consequences of replication stress. Downregulated proteins (Figure 2A; PB DNA repair) included, for example, the ATRX, whose mutation in multiple cancers suggests a role in tumor induction and progression.^75^ Additionally, ATRX plays a role in genomic stability and may prevent replication stress.^76^ Furthermore, BRIP1 (also known as BACH1) is a DNA helicase which acts as a potential tumor suppressor.^77^ BRIP1 preserves the integrity of the genome through its interaction with the BRCT domain of BRCA1.^78^ BRIP1 deficient cells display homologous recombination (HR) after DNA double-stranded breaks (DSBs).^79^ Further, downregulated proteins were the Polymerase delta 1 (POLD1), a catalytic and proofreading subunit of the human DNA polymerase delta (Polδ)^80^^,^^81^ as well as RECQl5, which plays a role in the repair of double-strand breaks,^82^ i.e. suppresses oncogenic JAK2-induced replication stress and genomic instability^83^ and supports the processing of stalled replication forks.^84^ Upregulated processes derived from the bafilomycin signature (Figure 2B) included apoptosis, the Notch signaling pathway, cell adhesion, differentiation, and glycolysis, the latter coherent with the overall mitochondrial loss-of-function signature. Furthermore, an upregulation of pathways connected to phosphorylation was found (Figure S1). Since mitochondrial dysfunction may be related to oxidative stress, the bafilomycin-induced signature was compared with the one triggered by H_2_O_2_ (Figures 2C–2E). A principal component analysis (PCA, Figure 2D) revealed three clearly separated clusters corresponding to the controls, bafilomycin and H_2_O_2_ treated cells respectively. Only a small number of proteins were regulated upon H_2_O_2_ treatment (4 down and 4 up, nuclear fractions), compared to bafilomycin (323 regulated proteins); from these proteins ACOT8, POTEJ, FOXA1, TINF2, and ANKRD30A were regulated in both conditions (Figure 2E). Of those FOXA1 was previously connected to a ROS response, namely H_2_O_2_ induced an increase in FOXA1 expression promoting apoptosis.^85^ Yet, FOXA1 expression can also be increased independently from ROS and it was previously described that FOXA1 level increased following the inhibition of lysosomal degradation using bafilomycin, or chloriquine.^86^ TINF2 is a regulator of telomer length and therefore potentially connected to DNA maintenance,^87^ its presence in the nuclear extract of the bafilomycin treated cells may on the other hand be explained by its known interaction with mitochondria.^88^

### Concentration dependent effects of bafilomycin on mitochondrial network and cell stiffness in T24 bladder cancer cells

**Figure 3:**
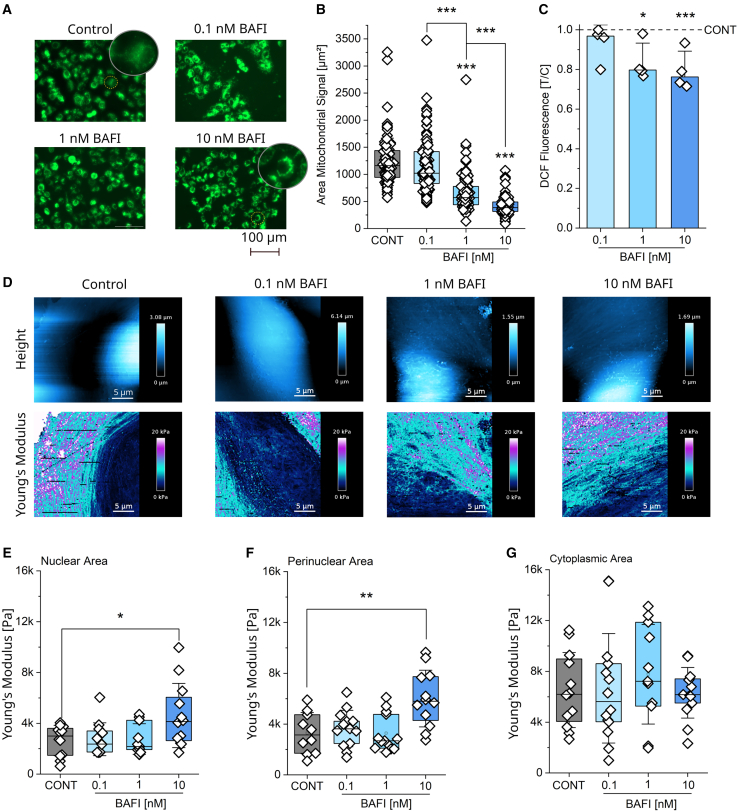
Short term incubation with bafilomycin and catalase changes the morphology of the mitochondrial network in absence of oxidative stress (A) Representative images of T24 cells stained with MitoTracker mitochondrial network (green) in control cells and following incubation with bafilomycin (0.1-1-10 nM, 4 h). Scale bar 100 μm. (B) Quantification of the mitochondrial network area (μm^2^), of control cells and cells treated with bafilomycin (0.1-1-10 nM). Results are shown as boxplots, whiskers represent SD, and boxes represent the range from 25 to 75 percentage. For the quantification of the areas *n* ≥ 70 cells were evaluated. For the statistical evaluation of the results One-Way ANOVA test with Fisher LSD was performed (∗∗∗*p* < 0.001). A comparison of the mitochondrial signal intensity can be found in Figure S2. (C) Quantification of the oxidative stress as DCF fluorescence in control cells and cells treated with bafilomycin (0.1-1-10 nM, 4 h). *N* = 4 biological replicates. Results for treated cells are shown as bar charts, while the control is depicted as a line, and whiskers represent SD. For the statistical evaluation of the results, one-way ANOVA test with Fisher LSD for means comparison was performed (∗*p* < 0.05, ∗∗∗*p* < 0.001). (D) Representative AFM maps show height and Young’s modulus, of control cells and cells incubated with bafilomycin (0.1-1-10 nM, 4 h). Scale bar 5 μm. (E) Quantification of the Young’s modulus in the nuclear area. (F) Quantification of the Young’s modulus in the perinuclear area. (G) Quantification of the Young’s modulus in the cytoplasmic area. Results (Figures 3E–3G) are shown as boxplots, whiskers represent SD, and boxes represent the range from 25 to 75 percentage. For the statistical evaluation of the results, one-way ANOVA test with Fisher’s LSD for means comparison was performed (∗*p* < 0.05, ∗∗*p* < 0.01). *n* ≥ 10 cells were quantified per condition. (*n* ≥ 3 biological replicates).

As proteome data supported the presence of DNA damage in relation to perinuclear organelle clustering, additional experiments were performed to start isolating the contribution of physical (deformation) and chemical (ROS) stress derived from the mitochondrial rearrangement. Indeed, with the mitochondrial accumulation in the nuclear region, it is possible that ROS stemming from the organelles could be responsible for the DNA-stress signature highlighted in the proteome analysis. Firstly, incubation time was reduced to 4 h to limit secondary effects related to gene transcription and/or unspecific responses arising from the prolonged inhibition of the autophagic cascade. Morphological rearrangement of the mitochondrial network was visible even after short time exposure (Figure 3A). Mitochondrial shift toward the nuclei was measurable, and the quantification returned a concentration dependent decrease of the networks area (0.1-1-10 nM BAFI; Figure 3B), while the signal intensity of the network remained constant (Figure S2). In parallel, intracellular ROS was monitored via DCF assay, as in the literature an increase of the oxidative stress marker dityrosine upon bafilomycin treatment is reported for RPE-1 cells.^89^ However, in T24 cells, DCF-assay returned a signature of contained oxidative stress with the fluorescence signal rather decreasing parallel to the mitochondria perinuclear clustering (Figures 3B and 3C).

Building on our previous findings that a relocalization of the organelles (ER) can alter the biophysical landscape of the cell,^52^ atomic force microscopy (AFM) measurements of cell stiffness as Young’s modulus were performed on cells incubated with 0.1-1-10 nM bafilomycin for 4 h (Figure 3D). By selecting ROIs within different intracellular compartments and quantifying the corresponding areas in the AFM maps, selective stiffness measurement of the nuclear, perinuclear, and cytoplasmic areas was possible. For each measured ROI, the median of all Young’s moduli calculated for the individual force curves was pooled and used for further evaluations. Parallel to the mitochondrial relocation, bafilomycin triggered a concentration dependent increase in intracellular stiffness, culminating in a significant increase of the Young’s modulus in both the nuclear (Figure 3E) and perinuclear regions (Figure 3F) upon the application of 10 nM bafilomycin (4 h). In the cell’s cytoplasmic area, no significant changes in stiffness were observed (Figure 3G).

### Contribution of oxidative stress to the effects of bafilomycin on mitochondrial network and cell stiffness in T24 bladder cancer cells

**Figure 4:**
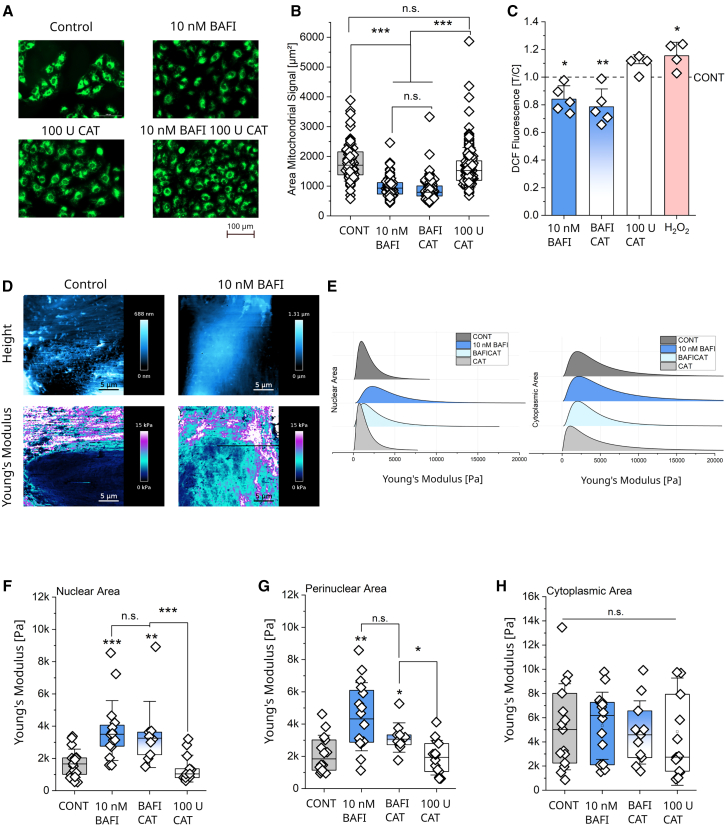
Effects of the mitochondrial rearrangement on cell stiffness and nuclear morphology (A) Representative images of mitochondrial network (green) in T24 control cells and cells incubated with bafilomycin (10 nM), catalase (100 U/mL), and the combination of the two. Scale bar 100 μm. (B) Quantification of the mitochondrial network area (μm^2^), of control cells and cells treated with bafilomycin (10 nM), catalase (100 U/mL), and the combination of the two. For the quantification of the areas, *n* ≥ 80 cells were evaluated. Three biological replicates were performed. Results are shown as boxplots, whiskers represent SD, and boxes represent the range from 25 to 75 percentage. For the statistical evaluation of the results, Student’s t test was performed (∗∗∗*p* < 0.001). (C) Quantification of the oxidative stress as DCF fluorescence in control cells and cells treated with bafilomycin (10 nM), catalase (100 U/mL), and the combination of the two, as well as H_2_O_2_ (0.5 mM) for 4 h. For the DCF assay, four biological replicates were performed. Results are normalized to the control. Results are shown as bar charts, whiskers represent SD, and boxes represent the range from 25 to 75 percentage. For the statistical evaluation of the results, Student’s t test was performed (∗*p* < 0.05, ∗∗*p* < 0.01). (D) Representative AFM maps show height and Young’s modulus of T24 controls and cells treated with 10 nM bafilomycin for 4 h. Scale bar 5 μm. Additional measurements of the cell area and AFM maps for remaining treatments (BAFI/CAT and CAT) can be found in Figures S3 and S4, respectively. (E) Distribution curves, composed of Young’s Moduli for all pixels/force curves quantified in the subcellular ROIs (Nuclear and Cytoplasm; *n* ≥ 10 cells), for control cells and cells treated with bafilomycin, catalase and the combination of the two for 4 h. (F) Quantification of the stiffness measured as Young’s modulus (Pa) in the nuclear area of T24 cells treated with bafilomycin (10 nM), catalase (100 U/mL), and the combination of the two for 4 h. (G) Quantification in the perinuclear area. (H) Quantification in the cytoplasmic area. Results (Figures 4F–4H) are shown as boxplots, whiskers represent SD, and boxes represent the range from 25 to 75 percentage. For the statistical evaluation of the results, Mann-Whitney test was performed (∗*p* < 0.05, ∗∗*p* < 0.01, ∗∗∗*p* < 0.001). At least *n* ≥ 10 cells were measured per condition, and at least three biological replicates were performed.

In order to further reduce potential interference related to intracellular ROS (i.e. stemming from H_2_O_2_), experiments were repeated, including the coincubation with the antioxidant enzyme catalase^90^^,^^91^ (CAT, Figure 4A). The presence of the antioxidant did not modify the response profile of bafilomycin, as the compound alone or in combination with catalase maintained the capacity to reduce the mitochondrial network footprint, while catalase alone returned no alterations in comparison to the controls (Figure 4B). Bafilomycin and the coincubation with catalase reduced the DCF signal to a similar extent (Figure 4C). Total cell area remained unchanged for all treatments (Figure S3), limiting the possibility that the organelle relocalization could be an artifact of cell shrinkage.

Proceeding with the characterization of the cell biomechanical properties, AFM experiments were conducted, including catalase in the experimental layout. Cells were incubated for 4 h with 10 nM bafilomycin, 100 U/mL catalase, and the combination of the two, and cell height and Young’s modulus were measured (Figures 4D, 4E, and S4). Aligning with the morphometric description of the mitochondrial clustering in the nuclear area (Figure 4F), both bafilomycin and the bafilomycin-catalase coincubation resulted in a stiffness increase. Control cells had a median Young’s modulus in the nuclear area of 1658 Pa, while the bafilomycin treated cells had values of 3497 Pa and bafilomycin and catalase treated cells 3256 Pa. The same pattern could also be found in the perinuclear area (Figure 4G), albeit the response of the treatment bafilomycin-catalase was slightly less efficient than the application of bafilomycin alone. In the cytoplasmic area no difference between the treatments was observed (Figure 4H).

### Effect of bafilomycin on the nuclear morphology and genotoxic damage in T24 bladder cancer cells

**Figure 5:**
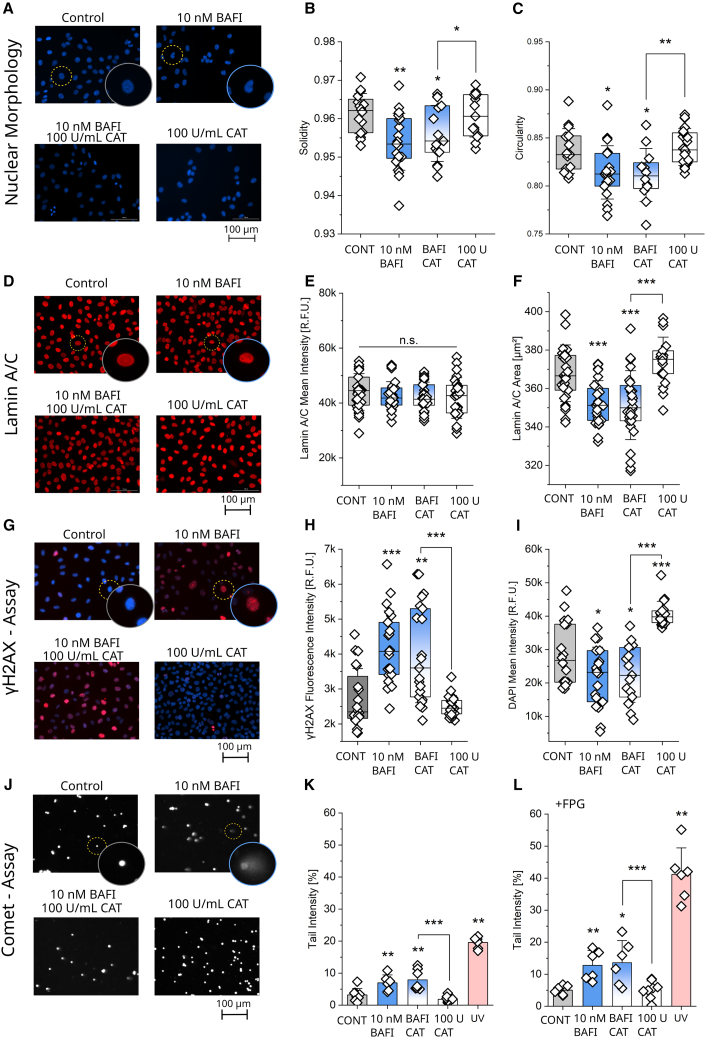
DNA damage signature following short term bafilomycin incubation (A) Representative images of cell nuclei with DAPI (blue) in T24 control cells and cells treated with bafilomycin (10 nM), catalase (100 U/mL), and the combination of the two for 4 h. Scale bar 100μm. (B) Quantification of the morphological marker solidity of the nuclei. (C) Quantification of the morphological marker circularity of the nuclei. For the analysis of nuclear morphology (Figures 5B and 5C), at least *n* > 12 optical fields were taken from at least three biological replicates. Additional parameters of nuclear morphology (Aspect Ratio, Area, and Roundness) can be found in Figure S5. A quantification of the actin signal can be found in Figure S6. (D) Representative images of the Lamin A/C staining (red) treated with bafilomycin (10 nM), catalase (100 U/mL), and the combination of the two for 4 h. Scale bar 100 μm. (E) Quantification of Lamin A/C intensity in T24 cells. (F) Quantification of Lamin A/C area in T24 cells. Results (Figures 5E and 5F) taken from *n* = 27 optical fields. (G) Representative images of the γH2AX (red) and DAPI (blue) signal in T24 cells treated with bafilomycin (10 nM), catalase (100 U/mL), and the combination of the two for 4 h. Scale bar 100 μm. (H) Quantification of the γH2AX intensity (R.F.U.) in the nuclear area. (I) Quantification of the DAPI intensity (R.F.U.). For the quantification of the γH2AX and DAPI intensity (R.F.U), at least 17 optical fields were evaluated, taken from at least three biological replicates (Figures 5H and 5I). (J) Representative images of the comet assay (DNA stained with ethidium bromide depicted in white) in T24 cells treated with bafilomycin (10 nM), catalase (100 U/mL) and the combination of the two. Scale bar 100 μm. (K) Quantification of the tail intensity of T24 cells. (L) Quantification of the tail intensity of T24 cells treated for 30 min with FPG. For the comet assay, at least three biological replicates were quantified. At least *n* = 6 patches were quantified (average of 50 cells per gel patch). A statistical comparison between the assay performed with and without FPG treatment can be found in Figure S7. Results (Figure 5) are shown as boxplots or bar charts (comets), whiskers represent SD, and boxes represent the range from 25 to 75 percentage. For the statistical evaluation of the results, Student’s *t*-Test, or Mann-Whitney Test (Comets) was performed (∗*p* < 0.05, ∗∗*p* < 0.01, ∗∗∗*p* < 0.001).

Combining the data of the mitochondrial profiling with the AFM measurements, it was possible to postulate that the intracellular shifts of the mitochondria could account for nuclear deformation. To investigate this, nuclear morphology was assessed using DAPI masks for reference (Figure 5A). For the shape descriptors solidity (Figure 5B) and circularity (Figure 5C) both bafilomycin alone and the combination with catalase reduced the two parameters, describing a distortion of the evenly round shape of the nuclei which is visible in control cells. Catalase effectively decreased the aspect ratio and increased the roundness of the nuclear signal, indicating changes toward a more circular shape (Figure S5), yet its coincubation with bafilomycin did not modulate the response profile of the autophagy inhibitor. Nuclear size measured as the total area of the DAPI signal remained unchanged among the treatments (Figure S5). In response to physical stress, the nuclear lamina acts as an important protective envelope and stabilizer for the nucleus.^92^^,^^93^ To investigate this, Lamin A/C was stained (Figure 5D) after incubation with bafilomycin in the presence or absence of catalase: the quantification of the signal intensity (20× magnification) returned no significant changes (Figure 5E). However, the area covered by the Lamin A/C signal was reduced in the cells incubated with 10 nM bafilomycin for 4 h. This effect also remained when catalase was co-incubated with the autophagy inhibitor (Figure 5F). Catalase alone had no effect, retracing the results of the nuclear morphology analysis. The actin cytoskeleton is another important structural component of the intracellular architecture and is directly involved in nuclear mechanotransduction and stability via its connection to the linker of nucleoskeleton and cytoskeleton (LINC) complex.^31^^,^^94^ Therefore, a potential contribution to the observed effects was considered in the present study. Yet, phalloidin staining of actin revealed no significant alteration in the signal in the chosen conditions (Figure S6), suggesting a limited contribution of the actin cytoskeleton in the adjustment of nuclear morphology.

In order to verify if the morphometric changes in the nuclear architecture could be connected to DNA damage, as previously described during confined migration for nuclear envelope rupture^7^ and increased replication stress,^8^ two assays were performed. Firstly, the γH2AX assay to detect the phosphorylated form of H2AX, namely γH2AX, which is formed in response to strand breaks (Figure 5G). Secondly the alkaline comet assay,^95^^,^^96^ which detects the sum of single and double-strand breaks using single cell electrophoreses. By incorporating the formamidopyrimidine-DNA glycosylase (FPG) enzyme in the experimental approach, specific damaged bases can be identified and converted into strand breaks, which are detectable using the comet assay.^97^ In the γH2AX assay (Figure 5G) both bafilomycin and bafilomycin/catalase treated cells showed an increase in the biomarker intensity in the nuclear region compared to the control and catalase alone (Figure 5H). The intensity of the 4′,6-diamidino-2-phenylindol (DAPI) signal mirrored this effect, decreasing in the bafilomycin containing conditions and increasing in cells treated with catalase (Figure 5I). Increased DAPI signal was previously connected to chromatin condensation in apoptotic cells.^98^ A similar trend was observed in the comet assay (Figure 5J), where in the absence of FPG treatment (Figure 5K) both bafilomycin and the cells treated with the combination of bafilomycin and catalase showed an increase in tail intensity of almost double the values compared to the controls and catalase. The same pattern was observed with the FPG-modified comet assay (Figure 5L). Here, all conditions displayed higher values than the standard assay (Figure S7). Regarding this general increase, a specific contribution of (oxidative) base damage to the mechanism of bafilomycin seemed unlikely at this point, as it should result in a selective increase of the tail intensity in the bafilomycin treatments in the FPG-modified assay.

### Dependency between mitochondrial rearrangement and genotoxic damage in T24 bladder cancer cells

**Figure 6:**
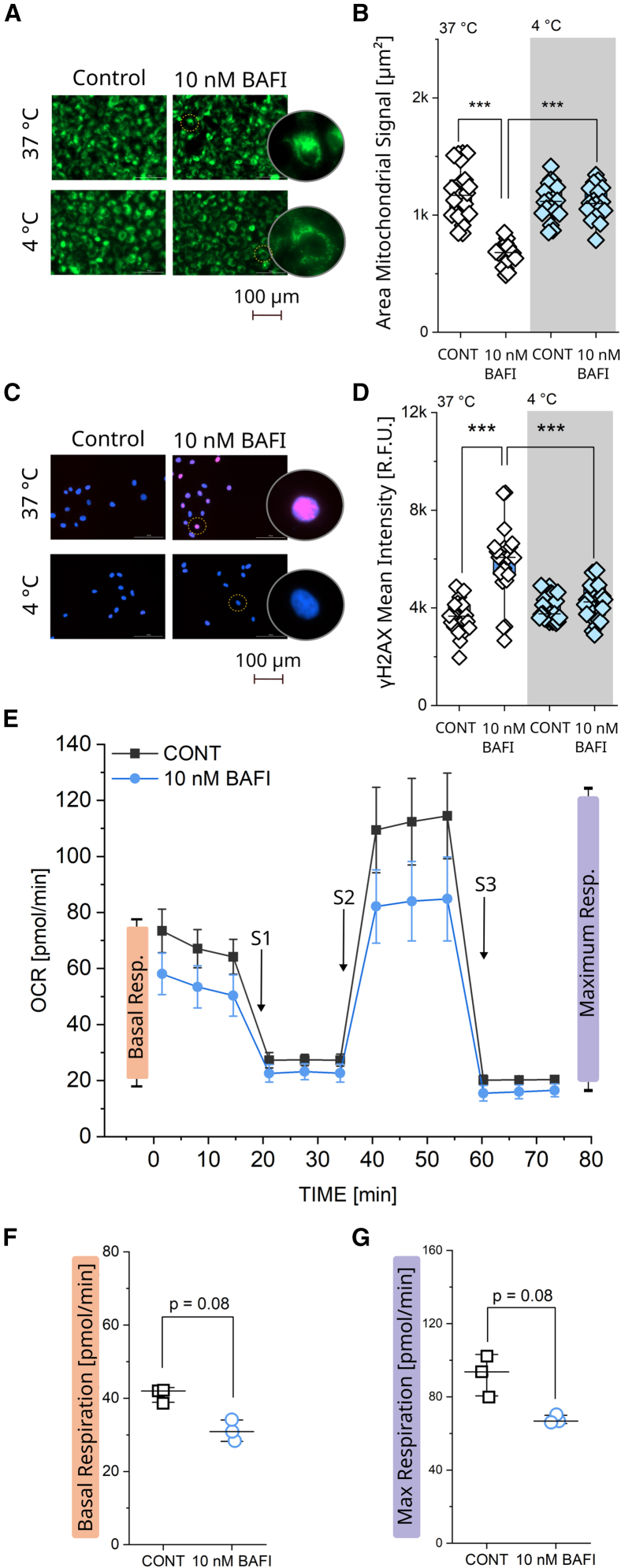
Profiling of mitochondrial function, temperature dependency and Seahorse assay (A) Representative images of mitochondrial network (green) in T24 cells incubated at 37°C and 4°C control cells with and without bafilomycin (10 nM) treatment for 4 h. Scale bar 100 μm. (B) Quantification of the mitochondrial area in T24 cells, average area of mitochondrial signal per optical field (*n* ≥ 18 optical fields). Results are shown as boxplots, whiskers represent SD, and boxes represent the range from 25 to 75 percentage. For the statistical evaluation of the results, Student’s t test was performed (∗*p* < 0.05, ∗∗*p* < 0.01, ∗∗∗*p* < 0.001). (C) Representative images of γH2AX (red) and DAPI (blue) signal in T24 cells, following incubation at 37°C and 4°C with bafilomycin (10 nM) for 4 h. Scale bar 100 μm. (D) Quantification of the γH2AX signal inside the nuclear area of T24 cells, average area of mitochondrial signal per optical field (*n* ≥ 18 optical fields). Results are shown as boxplots, whiskers represent SD, and boxes represent the range from 25 to 75 percentage. For the statistical evaluation of the results, Student’s t test was performed (∗*p* < 0.05, ∗∗*p* < 0.01, ∗∗∗*p* < 0.001). (E) Results of the Seahorse Assay of T24 control cells vs. cells pretreated with 10 nM bafilomycin for 4 h. Shown is the time dependent change in oxygen consumption rate (OCR) over time. Following the measurement of the basal respiration, the first stimulus 0.5 μM oligomycin (S1) was added, followed by 1.5 μM CCCP (S2), and finally 0.5 μM antimycin A and rotenone (S3). (F) Quantification of basal respiration (initial OCR minus the non-mitochondrial respiration (after AA/ROT)) was determined. (G) Quantification of the maximum respiration (OCR after CCCP addition minus OCR after AA/ROT addition). Results (Figures 6F and 6G) are shown as individual datapoints, whiskers represent SD and boxes represent the range from 25 to 75 percentage. In total 9 technical replicates per condition were performed (*n* = 3 biological replicates) and averages per biological replicate were used for statistical evaluation. (Mann-Whitney test (∗*p* < 0.05, ∗∗*p* < 0.01, ∗∗∗*p* < 0.001)).

In order to further explore the dependency between mitochondrial relocation and genotoxic damage, experiments were repeated at 4°C during the 4 h bafilomycin incubation as the low temperature would reduce energy production^99^^,^^100^ and potentially slow or prevent the movement of the organelles without changing the chemical composition of the incubation layout (Figure 6A).

In this case, experiments performed at 37°C served as control and retraced the results of the previous assays (Figure 6B), namely, a reduction of the mitochondrial area could be measured upon exposure to 10 nM bafilomycin. Yet at 4°C, no rearrangement of the mitochondrial network was observable (Figure 6B). Aligning with the response of the mitochondrial network, the γH2AX signal (Figure 6C) only increased in cells treated with bafilomycin at 37°C (Figure 6D). In controls at 4°C as well as in cells treated with bafilomycin at the reduced temperature no change in the γH2AX signal could be observed, when compared to the 37°C controls.

The rearrangement of the whole mitochondrial network represents a significant alteration of the cellular landscape, which might impact energy production and cell viability, as previously described for bafilomycin.^101^^,^^102^^,^^103^ In order to verify the mitochondrial functionality during the exposure to bafilomycin, Seahorse XF assay was performed (4 h, Figure 6E).^104^^,^^105^ The bafilomycin treated cells displayed a tendency toward a decrease in basal respiration (Figure 6F). After the addition of oligomycin (S1), respiration fell to a similar level for controls and treated cells. Upon the application of the uncoupler carbonyl cyanide *m*-chlorophenylhydrazone (CCCP, S2), a slightly lower maximum oxygen consumption rate (OCR) was measured in the bafilomycin treated cells in comparison to controls (Figure 6G). Finally, Antimycin A and Rotenone treatments (S3) were added, resulting in a similar residual non-mitochondrial respiration for both controls and treated cells. Aligning with the proteome signature measured after 24 h incubation (Figure 2A, decrease biological processes connected to the “Respiratory Chain and the Electron Transport”) it seems that some potential dysfunction of the mitochondrial respiration may start in the first hours of bafilomycin incubation, although this was maintained moderate within 4 h of experimental time, compared to experiments found in literature for 24 h incubation with bafilomycin.^101^

### Effects of bafilomycin on SK-OV-3 ovarian cancer and HCT 116 colon cancer cell line

**Figure 7:**
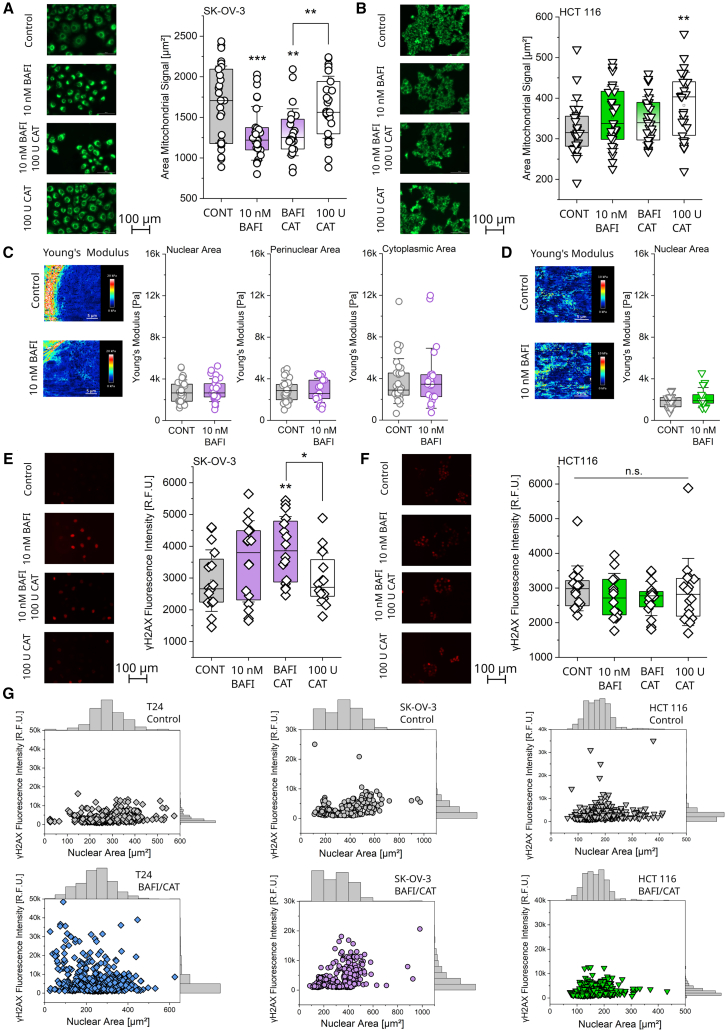
Bafilomycin induces mitochondrial rearrangement in SK-OV-3 but not HCT 116 cells (A) Representative images and quantification of the mitochondrial network (green) in SK-OV-3 cells. (B) Representative images and quantification of the mitochondrial network (green) in HCT 116 cells, respectively. Scale bar 100 μm. Staining of live cells was performed with MitoTracker. (Figures 7A and 7B) 5 cells were evaluated per optical field, and the average was formed. At least *n* ≥ 24 optical fields per condition were evaluated, in three biological replicates. Quantification of the lysosomal signal can be found in Figure S8. (C) Representative AFM stiffness (Young’s modulus) maps and measurement of cell stiffness in SK-OV-3 cells (nuclear, perinuclear, and cytoplasmic area). (D) Representative AFM stiffness (Young’s modulus) maps and measurement of cell stiffness in HCT-116 cells (Nuclear area). (Figures 7C and 7D) At least *n* ≥ 15 cells per condition, were measured taken from three biological replicates. Scale bar 5 μm. An additional comparison of cell stiffness in control T24, SK-OV-3, and HCT116 cells can be found in Figure S9. (E) Representative images and quantification of the γH2AX assay (red) in SK-OV-3. (F) and HCT 116 cells, respectively. At least *n* ≥ 16 optical fields were quantified, and the average mean intensity per cell for the image was determined. Cells taken from 4 biological replicates. Scale bar 100 μm. (Figures 7A–7F) Results are shown as boxplots, whiskers represent SD and boxes represent the range from 25 to 75 percentage. For the statistical evaluation of the results, one-way ANOVA with Fisher LSD test or Mann-Whitney Test (AFM, γH2AX) was performed (∗*p* < 0.05, ∗∗*p* < 0.01, ∗∗∗*p* < 0.001). Additional quantifications of nuclear morphology can be found in Figure S10. (G) Correlation between the γH2AX signal (y axis) and the area of the nuclei (x axis) for cells treated with bafilomycin and catalase versus control cells. All individual cells taken from the γH2AX and nuclear morphology quantification (Figures 7E, 7F, and S10) were used for these quantifications.

In order to investigate if the mechanism of bafilomycin-induced intracellular rearrangement and genotoxic damage is unique to bladder cells, the key experiments were repeated using other cell lines. Expanding the represented tissues, the ovarian cell line SK-OV-3 and the colon cancer cell line HCT 116 were included. Apart from having a sensitive mechanosensory apparatus,^106^^,^^107^ these cells were selected because they are cultivated in the same medium as the T24, therefore avoiding potential solubility issues and ensuring cells being exposed to the same nutrients and supplements. Even though the fluorescence intensities (dependent on lysosomal acidification) showed cell-line specific responses among the controls for LysoSensor and LysoTracker stainings, the activity of bafilomycin returned the same signature in all three cell lines (Figure S8).^40^^,^^68^ Building on this, in SK-OV-3 cells (Figure 7A; 4 h incubation), the area of the mitochondrial network was significantly reduced by both bafilomycin and its coincubation with catalase. The HCT 116 cells (Figure 7B) did not respond to the bafilomycin treatment and only increased their mitochondrial area after 4 h incubation with catalase.

Cell stiffness, measured as Young’s modulus, remained unaltered in both cell lines (SK-OV-3 Figure 7C and HCT 116 Figure 7D). For the HCT 116 cells, due to their small size, no selective measurement of the perinuclear and cytoplasmic area was reliably possible. Of note, the stiffness of the three cell lines was different even in control conditions, with SK-OV-3 cells’ stiffness being significantly higher than that of T24 (nuclear, perinuclear area) and HCT 116 (nuclear area) cells (Figure S9). Additionally, the nuclear morphology of SK-OV-3 and HCT 116 cells was quantified. For the two parameters, solidity and circularity, altered in the T24 cells upon bafilomycin treatment, only circularity in the SK-OV-3 cells resembled the response profile of the bladder cells (Figure S10). Retracing the rearrangement of the mitochondrial network, the γH2AX signal was increased only in the bafilomycin treated SK-OV-3 cells (Figure 7E), and albeit showing a clear trend, this was significant only for the combination of bafilomycin and catalase. The γH2AX signal in the HCT 116 cells was not affected by the treatments (Figure 7F). Finally, the correlation of the nuclear area determined by the DAPI signal to the intensity of the γH2AX signal, further highlighted the response profile of the different cell lines (Figure 7G). In control conditions, all three cell types presented uniform γH2AX signals through the distribution of the morphometric descriptor. For the T24 and SK-OV-3 cells treated with bafilomycin and catalase it is possible to observe that an increase of the γH2AX signal is accompanied by a reduction of the nuclear area. This indicates that a decrease in the nuclear size positively correlates with the intensity of the marker for double-strand breaks. For the HCT 116 cell line, the signal pattern remained unchanged between controls and treated cells.

**Figure 8:**
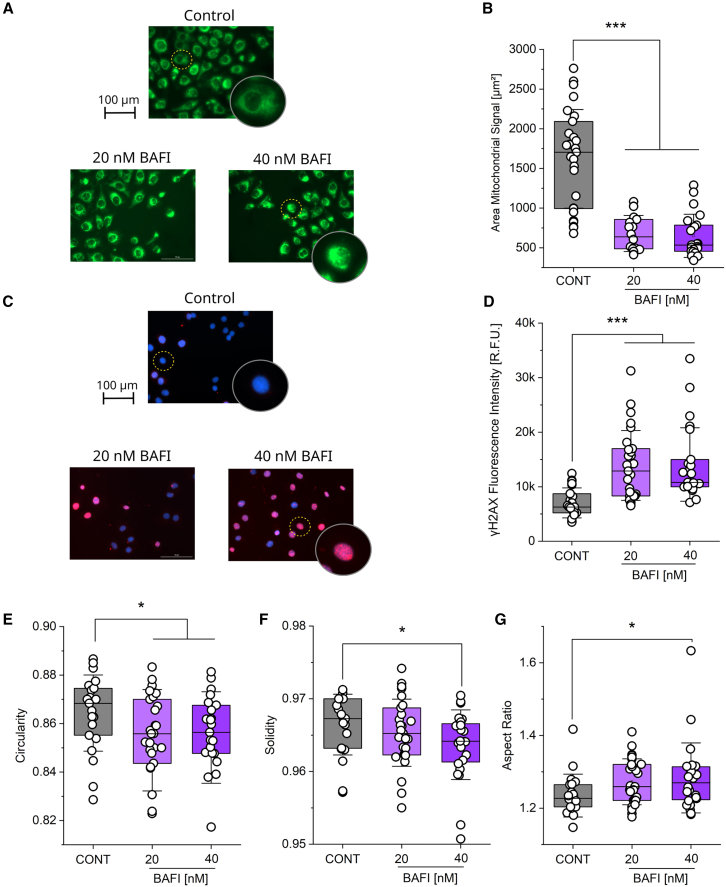
Bafilomycin in higher concentrations induces mitochondrial rearrangement, nuclear and perinuclear stiffness increase, and γH2AX increase in SK-OV-3 cells (A) Representative images of MitoTracker stained mitochondrial network (green) in the SK-OV-3 following 4 h 20 and 40 nM bafilomycin treatment. Scale bar 100 μm. (B) Quantification of the signal area of the mitochondrial network in the SK-OV-3 following 4 h 20 and 40 nM bafilomycin treatment. For each condition the average per 5 cells per optical field (*n* ≥ 18 optical fields) was calculated. (C) Representative images of γH2AX (red) and DAPI (blue) stained SK-OV-3 cells following 4 h 20 and 40 nM bafilomycin treatment. Scale bar 100 μm. (D) Quantification of the nuclear γH2AX signal in SK-OV-3 following 4 h 20 and 40 nM bafilomycin treatment. For each condition, at least, *n* ≥ 21 optical fields (means per optical field) were evaluated. (E) Quantification of the nuclear morphology marker “Circularity.” (F) Quantification of the nuclear morphology marker “Aspect Ratio.” (G) Quantification of the nuclear morphology marker “Solidity.” For each condition (Figures 8E–G) at least, *n* ≥ 21 optical fields (means per optical field) were evaluated. Results (Figure 8) are shown as boxplots, whiskers represent SD and boxes represent the range from 25 to 75 percentage. For the statistical evaluation one-way ANOVA test with Fisher LSD was performed (∗*p* < 0.05, ∗∗*p* < 0.01, ∗∗∗*p* < 0.001). Experiments were performed in 3 biological replicates.

While the incubation of SK-OV-3 cells with 10 nM bafilomycin did not increase the γH2AX signal significantly, a similar signature as in the T24 cells could be observed in the previously discussed experiments (Figures 5 and 7). Hypothesizing that the lack of a significant response of the SK-OV-3 could be related to a different sensitivity to bafilomycin of the SK-OV-3 cell line compared to the T24, further experiments were performed with increased concentrations of bafilomycin (20, 40 nM, 4 h). Aligning with the previous results, mitochondrial area decreased at both tested concentrations (Figures 8A and 8B). γH2AX staining was performed, and its nuclear signal significantly increased in SK-OV-3 cells treated with both 20 and 40 nM bafilomycin for 4 h (Figures 8C and 8D). Furthermore, nuclear morphology was quantified; the parameters circularity (Figure 8E, 20 and 40 nM bafilomycin) and solidity (Figure 8F, 40 nM bafilomycin) were significantly reduced, aligning completely with the data collected in the T24 cell line (Figures 5B and 5C). In addition, the parameter aspect ratio was increased in SK-OV-3 cells treated with bafilomycin (Figure 8G, 40 nM).

### Effects of bafilomycin on Lamin A/C morphology and pan-phosphorylation signature

**Figure 9:**
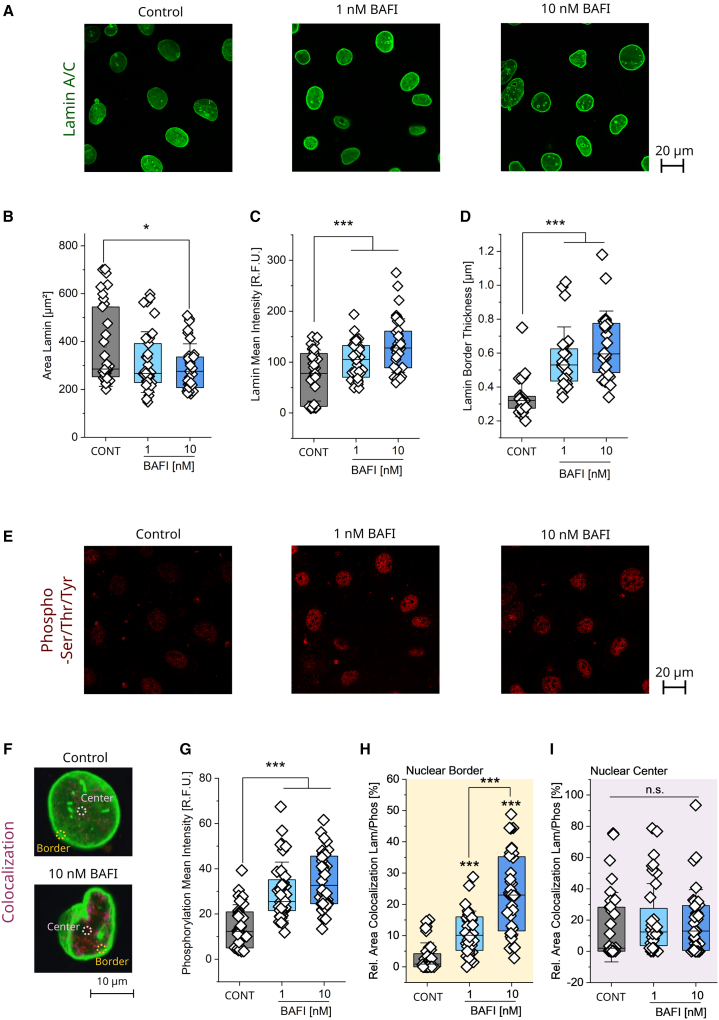
Bafilomycin changes phosphorylation and Lamin A/C signal distribution and colocalization (A) Representative images of T24 cells stained for Lamin A/C (green) following 4 h 1 and 10 nM bafilomycin treatment. Scale bar 20 μm. (B) Quantification of the Lamin A/C area in T24 cells. (C) Mean intensity per cell of the Lamin A/C signal. At least *n* ≥ 33 cells were evaluated taken from 12 optical fields (3 biological replicates) (Figures 9B and 9C). (D) Quantification of Lamin A/C border thickness. Thickness of the Lamin A/C signal was measured at 10 locations around two cells per optical field. Mean per cell was formed *n* = 24 cells per condition were evaluated (3 biological replicates). (E) Representative images of T24 cells stained for phosphoserine/threonine/tyrosine (red) following 4 h 1 and 10 nM bafilomycin treatment. (F) Representative control and bafilomycin treated cells (magnified and cut out), colocalization (pink) between Lamin A/C (green) and phosphoserine/threonine/tyrosine (red). Highlighted are the areas selected for quantification, i.e., border of the lamina and the center. Scale bar 10 μm. (G) Mean intensity per cell of the phosphoserine/threonine/tyrosine signal. At least *n* ≥ 33 cells were evaluated from 12 optical fields (3 biological replicates). (H) Colocalization inside ROIs in the border of the Lamin A/C signal. Quantification of Lamin A/C and phosphorylation colocalization (Signal intensity threshold 75 R.F.U. for Lamin A/C and 50 R.F.U. for phosphorylation). (I) Colocalization in ROIs selected in the center of the Lamin A/C signal area. *N* ≥ 28 ROIs were investigated from 12 optical fields (3 biological replicates). (Figures 9H and 9I) For the statistical evaluation of the results, one way ANOVA tests with Fisher LSD were performed (∗*p* < 0.05, ∗∗*p* < 0.01, ∗∗∗*p* < 0.001). Quantification of the colocalization in the full optical fields can be found in Figure S11.

Albeit with cell line specific responses, the data collected pointed toward a central role of nuclear deformation in the response to bafilomycin (Figures 5, 7, and 8). Starting from the observation that the Lamin A/C has altered appearance in T24 cells following bafilomycin treatment (Figures 5E and 5F), this nucleoskeletal element was investigated in more detail using confocal microscopy (Figure 9A). Aligning with low resolution imaging, confocal data confirmed that 10 nM bafilomycin is effective in reducing the size of the Lamin A/C area (Figure 9B) in T24 cells. Additionally, higher resolution images allowed the observation that both 1 and 10 nM bafilomycin increased the Lamin A/C signal intensity significantly (Figure 9C). It was possible to appreciate that Lamin morphology also visibly changed, forming a border/ring at the edge of the nuclear rim characterized by high fluorescence intensity. The quantification of thickness appearance revealed an increase following bafilomycin incubation (Figure 9D). Furthermore, as mechanical stability of Lamin A/C is dependent on post-translational modifications, such as the phosphorylation status, staining of Lamin A/C was accompanied by the immunofluorescence of the pan-phosphorylation events (Figures 9E and 9F).^108^ Overall, phosphorylation measured within the area was significantly increased (Figure 9G), which also might relate to the increase of the histone phosphorylation as described in Figure 5H. Focusing the quantification on the lamina border, even with the limitations related to a broad spectrum of the detected phosphorylation events, an increase in the colocalization of Lamin A/C and phosphorylation signals could be measured (Figures 9F and 9H). The colocalization of the two signals in the center (Figure 9I) as well as in the whole image (Figure S11) remained unchanged between control and treated cells.

## Discussion

The regulation of DNA integrity in response to cell deformation is vital for living cells. Despite this, relatively little is known about the underlying molecular mechanisms in comparison to the management of chemical-induced damage. A correlation between nuclear morphology and genotoxicity was already established when external pressure acts on the cell and its nucleus, for example, during the simulation of confined migration.^7^^,^^9^ The results of the present study support the existence of pathways culminating with DNA damage when mechanical stress originates intracellularly, namely when the deformation of the nucleus correlates with mitochondrial clustering.

The mitochondrial shuttling is a known cellular response mechanism and can be induced through cellular energetic needs, metabolic stress, or in response to mitotoxic chemical cues.^109^^,^^110^^,^^111^ In the present study, bafilomycin A1^112^ was used as a chemical trigger to induce the movement of mitochondria. In addition to the effect as an autophagy inhibitor, the compound modifies the mitochondrial network, as previously described through its function as an inhibitor of V-ATPase^113^ and as a potassium ionophor.^114^ Untargeted proteome signature described a clustering of the mitochondria in the perinuclear compartment with the enrichment of the mitochondrial proteins (Figure 1) and a downregulation of the proteins of the mitochondrial respiratory chain (Figure 2).

Additionally, the proteome signature provided indications that replication stress may occur as proteins connected to both cell division and DNA replication were downregulated. Replication stress can be a consequence of chemical DNA modification, such as adducts or lesions, following exposure to mutagens or increased ROS. The resulting modifications act as obstacles for the DNA replication machinery and may result in DNA damage and genomic instability.^115^^,^^116^^,^^117^ Besides these chemical modifications, physical factors can also interfere with DNA replication. Nuclear compression is a recently discovered source of DNA damage: forced changes in DNA conformation or hindering of the DNA unfolding at replication forks are among the potential mechanisms of this type of interference.^8^

Considering that both chemical and physical cues may contribute to the genotoxic effects observed in this study, several approaches were followed to start isolating the individual components. For bafilomycin, genotoxicity is rarely described^89^; therefore, a direct contribution of the compound seemed unlikely. Yet, the clustering of mitochondria in the perinuclear region could be associated with increased radical stress in close proximity of the DNA.^118^^,^^119^ A reduced incubation time was privileged to limit the effects related to prolonged autophagy inhibition, including among others the accumulation of aged and dysfunctional mitochondria as typically associated to defective mitophagy.^120^^,^^121^^,^^122^ In the experiments performed in T24 cells, the contribution of ROS seems to be limited, as indicated by the results of the DCF assay (Figure 3C) and the data obtained in presence of catalase (Figure 4).^90^^,^^91^ In this context, differences between cells incubated with bafilomycin and the combination of bafilomycin with the antioxidant enzyme catalase were marginal in most experimental settings. Further supporting the view that ROS species could have limited impact on the data collected in our model, previous studies have shown that H_2_O_2_ associated ROS rather reduces cell stiffness.^123^^,^^124^^,^^125^ Furthermore, studies performed on cells during confined migration showed that deformation induced DNA damage cannot be mitigated by the ROS scavenger N-acetyl cysteine (NAC).^8^ Of note, in bladder cells exposed to catalase we could observe a tendency toward lower stiffness values in the distribution of the AFM measurements. This behavior can be possibly related to the morphometric adaptation of the nuclei in response to the antioxidant incubation (Figures S5A and S5C). However, additional studies are needed to fully explore these aspects, including for example, the kinetic of ROS fluctuations^126^^,^^127^ and the potential contribution of ROS species other than H_2_O_2_.^128^^,^^129^

Further exploring the correlation between mitochondrial rearrangement and DNA damage, experiments were performed at reduced temperature (4°C). Low temperature coherently inhibited the movement of the mitochondria, as well as the γH2AX signal further supporting the view of a link between the two events (Figure 6). Importantly, bafilomycin can diffuse into the cell through its lipophilic nature (4.69 Log P_o/w_, SwissADME^130^), and a reduction of its activity can be ascribed to a temperature dependent variation of the diffusion coefficient through the cell membrane^131^ or attributed to a decrease of cell metabolic performance which ultimately affects active transport mechanisms and intracellular organelles dynamics.^132^^,^^133^^,^^134^

Following the experimental hypothesis of the article, it was described that the increased retrograde movement of the mitochondrial network as shown by live cell imaging (Figures 3 and 4), is associated with an adaptation of nuclear shape and biomechanical properties (Figures 3 and 4). This observation is of central importance, as the nucleus is recognized as a mechanosensor,^21^ helping for example migrating cells with the gauging of the microenvironment, allowing adjustment to matrix stiffness or to confined spaces.^135^^,^^136^^,^^137^^,^^138^ In our model, an increase in nuclear stiffness was observed in relation to alterations of the intracellular landscape and might be attributed to several mechanisms. It was previously described that the application of compression as for nuclear indentation experiments returns increased nuclear stiffness.^139^ In our case, it is possible to hypothesize that the measured increase of Young’s modulus (Figures 3E, 3F, 4F, and 4G) could be the response to the increased perinuclear clustering of organelles such as the mitochondria (Figures 3A, 3B, 4A, and 4B), as we previously described also for the ER relocation.^52^ One factor that could contribute to explaining the particular role of mitochondria in the context of nuclear deformation is the close tethering of the organelle to the nucleus through membrane contact sites. These sites are emerging as key players in cellular signaling, especially in the retrograde mitochondrial response, and could be connected to the prolonged presence of mitochondria in the perinuclear region.^63^^,^^140^ Furthermore, the ER is known to be in close connection with the mitochondria^60^^,^^61^^,^^62^^,^^141^ making it plausible that the two organelles could move together. This view would be supported by the proteome signature describing the enrichment of membranes, Golgi apparatus, mitochondria as well as ER proteins in the nuclear extracts (Figure 1I).

Mechanotransduction and inward-outward signals can also support biochemical and biomechanical feedback loops: for example stretch-induced deformation can support intracellular calcium increase leading to heterochromatin-driven nuclear softening offering protection to mechanical damage.^142^ A softer nucleus can deform more easily, whereas a stiffer organelle may be more brittle and prone to damage.^136^^,^^143^^,^^144^ Likewise, softening or even “fluidification” of chromatin can be seen as an intended defense against stretch since a stiffer structure may rupture.^142^

The nuclear lamina acts as a protective barrier supporting the nuclear envelope: the reduction of its area (Figures 5F and 9B) may be interpreted as a failure of this function. Of note, properties of Lamin A/C are dependent on its phosphorylation status, softening and increasing mobility with increased phosphorylation and stiffening with the reduced phosphorylation of the protein.^108^^,^^145^ Cells treated with bafilomycin displayed a significant change in their Lamin A/C signal, resulting in the formation of an increasingly thick Lamin A/C border (Figure 9D). Yet, it remains to be elucidated if this might be the result of a direct or indirect effect. Hypothetically, changes on the nuclear stiffness can be related to direct post translational modifications of Lamin A/C, such as phosphorylation or dephosphorylation.^108^^,^^145^ Additionally, increased Young’s modulus might be the consequence of the collapse of the nucleoskeleton as for the Lamin A/C becoming less resistant to deformation due to increased phosphorylation (Figure 9) and incapable of coping with the mechanical load of the perinuclear organelle clustering. Increased Young’s modulus and increased nuclear deformation were already described for cells with decreased Lamin B expression and stable Lamin A/C expression.^34^ Furthermore, it was previously described that the phosphorylation of Lamin was connected to a relocalization to the nucleoplasm^146^ providing an additional factor possibly contributing to the reduced area of the Lamin A/C signal (Figure 9). Obviously, the phosphorylation events might even relate to other intranuclear pathways.^147^ Hence, phosphorylated H2AX following bafilomycin treatment (Figures 5G and 5H) might also contribute to the nuclear increase in pan-phosphorylation signal (Figure 9G), yet the biomarker is rather known to distribute in evenly dispersed foci throughout the nucleus.^148^^,^^149^

Of note, nuclear ATP surges, as those related to increased presence of mitochondria in the perinuclear area, have also been connected to a protective effect, supporting DNA repair pathways.^150^ Yet, seahorse measurements infer for limited or no variation of respiratory function upon 4 h incubation (Figures 6E–G), but these mechanisms can possibly play a role over longer incubation periods, as for the data supported by the proteome analysis (Figures 1 and 2).^101^

Expanding the evaluation to other cell lines, we could observe coherent responses between the T24, HCT 116 and SK-OV-3 (Figures 7 and 8). Among the three cell lines, lysosomal signals (Figure S8) and nuclear stiffness (Figure S9) already differed in control conditions, supporting the view that adaptation potential to biochemical and biophysical triggers could align with the original phenotypes. However, whenever a significant increase of the γH2AX signal was visible (Figures 5, 6, 7, and 8), this was accompanied by the rearrangement of the mitochondrial network (Figures 3, 4, 6, 7, and 8). It is possible to imagine that this phenotypic heterogeneity could potentially relate to the physical one, depending also on the stiffness variations of the surrounding tissue, fostering differences between different cancer cell populations^151^^,^^152^^,^^153^ like those returned by the experiments performed with T24, HCT 116, and SK-OV-3 cell lines.

In conclusion, it was possible to demonstrate that a retrograde shift of the mitochondrial network following bafilomycin A1 incubation correlates with a signature of nuclear deformation and increased DNA damage in T24 and SK-OV-3 cells. The implications of this discovery are at least 2-fold; on one hand, unraveling these mechanisms may be exploited to target cancer cells specifically and to better grasp essential knowledge on tumor response to physical internal and external constraints. Additionally, the data collected in this study support the presence of pathophysiological and toxicological pathways related to the physical rearrangement of intracellular organelles. As these effects are observable without cytotoxicity^40^ or significant functional impairment (Figure 6E), these findings have great potential for the creation of models that allow the investigation of sub-acute to chronic insults and might even allow the description of detrimental effects related to long-term chemical exposures.

### Limitations of the study

Inherently to the *in vitro* approach followed for this study, several limitations can be identified. Clearly, the translatability of the findings to *in vivo* conditions remains an open question. Within the mechanistic approach, by analyzing individual cells and not cell collectives, this leaves unanswered the question of the potential translation of the observed mechanisms within the constraints of tissues. Additionally, by comparing bladder (T24), ovarian cancer (SK-OV-3), and intestinal cells (HCT 116) cell type heterogeneities emerged. If this could be related to the original morphometric features of the control cells, the biophysical properties displayed before treatments, or the basal autophagic/metabolic competence of the different cell types remains an open and intriguing research question. Along these lines, the present work describes data exclusively collected in cancer cells, whose genetic profile, stability and repair mechanisms are *per* definition modified in comparison to non-transformed counterparts. As far as the execution of the study is concerned, the role of ROS species not covered by our experimental approach cannot be excluded as a contributing factor, and further studies are needed to explore their potential role in the generation of genotoxic damage in addition to the nuclear deformation. Related to the intracellular mass shifts, the involvement of other organelles (i.e. ER) moving coherently with the mitochondrial network remains to be investigated. Additionally, the phosphorylation status of nuclear Lamins could not be measured directly leaving it open, if a change of the PTMs could be responsible for the changes in biomechanical properties.

## Resource availability

### Lead contact

Requests for further information and resources should be directed to and will be fulfilled by the lead contact, Giorgia Del Favero (giorgia.del.favero@univie.ac.at, Josef-Holaubek-Platz 2, 1090, Vienna, Austria).

### Materials availability

This study did not generate new unique materials.

### Data and code availability

• The datasets generated during this study will be made available upon reasonable request to the lead contact, Giorgia Del Favero (giorgia.del.favero@univie.ac.at), Josef-Holaubek-Platz 2, 1090, Vienna, Austria).
• This study did not generate any code.
• The mass spectrometry proteomics data have been deposited in the ProteomeXchange Consortium via the PRIDE^154^ partner repository with the dataset identifier PXD060036.

## Acknowledgments

This work was supported by the University of Vienna (Intramural Funding). Additionally, this research was funded in part by the 10.13039/501100002428Austrian Science Fund (FWF) [10.55776/P35822]. For open access purposes, the author has applied a CC BY public copyright license to any author accepted article version arising from this submission. Imaging workflows were supported by the core facility multimodal imaging (Faculty of Chemistry, University of Vienna, Vienna Life Science Instruments-VLSI). The authors are grateful to Roberto Cerbino for insightful and stimulating scientific discussion, to Sarah Younan, Endre Kiss, Florian Call, Vanessa Partsch, Janice Bergen, Martina Karasová, and Maliha Hossain for precious technical contributions and to Yasmin Borutzki for the advice during the Seahorse experiments.

## Author contributions

Conceptualization G.D.F., methodology G.D.F., F.C., A.B., C.G., and S.M.M.M., formal analysis: M.J. and A.B., investigation: G.D.F. and M.J., resources: G.D.F., D.M., F.C., C.G., and S.M.M.M.; data curation: G.D.F., A.B., and C.G., visualization: M.J. and G.D.F., supervision: G.D.F., writing-original draft: M.J. and G.D.F., writing-review and editing: all authors.

## Declaration of interests

The authors declare no competing interests.

## STAR★Methods

### Key resources table

**Table:** 

REAGENT or RESOURCE	SOURCE	IDENTIFIER
**Antibodies**		

Anti-Lamin A/C mouse Antibody	Santa-Cruz	REF: sc-376248; RRID: AB_10991536
Anti-phospho-Histone H2A.X (Ser139) mouse Antibody	Merck	REF: 05-636; RRID: AB_309864
Phosphoserine/threonine/tyrosine Polyclonal rabbit Antibody	Invitrogen	REF: 61-8300; RRID: AB_2533941
Alexa Fluor 488 donkey anti-mouse	Invitrogen	REF: A21202; RRID: AB_141607
Alexa Fluor 647 donkey anti-rabbit	Invitrogen	REF: A315573; RRID: AB_2687541
Alexa Fluor 647 donkey anti-mouse	Invitrogen	REF: A31571; RRID: AB_162542

**Chemicals, peptides, and recombinant proteins**		

Bafilomycin A1	Sigma-Aldrich	REF: SML-1661
Phalloidin Oregon Green® 488	Invitrogen	REF: 07466
LysoSensor™ DND-189	Invitrogen	REF: L7435
LysoTracker™	Invitrogen	REF: L7528
MitoTracker™	Invtrogen	REF: M7514
ROTH Roti®Mount FluorCare DAPI	Roth	REF: HP 20.1
Oligomycin	Sigma	REF: O4876-5MG
Carbonyl cyanide 3-chlorophenylhydrazone (CCCP)	Sigma	REF: C2759-1G
Antimycin A	Sigma	REF: A8674-25MG
Rotenone	Tocris bioscience	REF: 3616

**Deposited data**		

Proteome	This paper	Project accession: PXD060036

**Experimental models: Cell lines**		

T24 human bladder cancer cell line	ATCC	RRID: CVCL_0554
HCT 116 colon cancer cell line	ATCC	RRID: CVCL_0291
SK-OV-3 ovarian cancer cell line	ATCC	RRID: CVCL_0532

**Software and algorithms**		

ZEN 2012 Black Edition	Zeiss	N/A
ImageJ 1.54f	NIH	RRID: SCR_003070
GEN5 Microplate Reader and Imager Software Version 3.05	BioTek	RRID: SCR_017317
OriginPro2023b	OriginLab	RRID: SCR_014212
MaxQuant 1.6.17.0	Max Planck Institute of Biochemistry	Cox and Mann^155^
Perseus software (version 1.6.14.0)	Max Planck Institute of Biochemistry	RRID: SCR_015753

**Other**		

LSM710 laser scanning confocal microscope ELYRA PS.1 system	Zeiss	RRID: SCR_018063
Lionheart FX Automated microscope	BioTek	RRID: SCR_019744
Dionex Ultimate 3000 nano high performance liquid chromatography	Dionex	RRID: SCR_019840
timsTOF Pro mass spectrometer	Bruker	N/A
Synergy H1 platereader	BioTek	RRID: SCR_019748
JPK NanoWizard® 4 XP atomic force microscope	Bruker	N/A
PFQNM-LC-A-CAL Pre-calibrated AFM tips	Bruker	N/A
PFQNM-LC-V2 Pre-calibrated AFM tips	Bruker	N/A
Seahorse XF HS Mini Analyzer	Agilent	N/A
MycoStrip® - Mycoplasma Testing Kit	InvivoGen	REF: rep-mys-20

### Experimental model and study participant details

#### Cell culture

T24 human bladder cancer (RRID: CVCL_0554, 81-year-old female), HCT 116 colon cancer (RRID: CVCL_0291, adult male) and SK-OV-3 ovarian cancer (RRID: CVCL_0532, 64-year-old female) cell lines were cultivated, and treatments were performed in Gibco™ McCoy’s (Modified) Medium 5A, supplemented with 10% fetal bovine serum and 1% penicillin/streptomycin. All cultivation and incubations were performed in humidified incubators (37°C, 5% CO_2_). Cell lines were not further authenticated. Cultures were tested for mycoplasma contamination using a MycoStrip® - Mycoplasma Testing Kit (InvivoGen). Sex and gender specific influences were not addressed in the scope of this study. Experiments were performed in at least three biological replicates (independent cell preparations), exact number of quantified cells, images and ROIs can be found in the respective figure legends.

### Method details

#### Compounds and materials

Bafilomycin A1 (REF: SML-1661) was purchased from Sigma-Aldrich (USA), stock solutions were prepared in DMSO and used to create incubation media with a dilution factor of 1000. Solvent controls were matched to the same DMSO concentration. Except for μ-Slide 8 Well ibiTreat (REF: 80.826, Ibidi GmbH, Gräfelfing, Germany) for the confocal experiments and tissue culture dishes (REF: 40 93040, TPP Techno Plastic Products AG, Switzerland) for the AFM experiments, all other cell culture materials were purchased from Sarstedt (Nümbrecht, Germany).

#### Live cell imaging

Cells were seeded and allowed to grow for 48 h. Incubation was performed for either 4 or 24 h (Bafilomycin 0.1-1-10 nM, Catalase 100 U/mL), after which cells were washed with warm Live Cell Imaging Solution (LCIS) Invitrogen (A59688DJ), and incubated for 15 minutes with 1:1000 MitoTracker™, or 1:1000 LysoSensor™ and 1:1000 LysoTracker™.^156^ After a washing step with LCIS, cells were imaged in LCIS. After the incubation the imaging was performed using a Zeiss LSM 710 laser scanning confocal microscope (ELYRA PS.1 system, RRID: SCR_018063) with a 63X/1.46 Plan-Apochromat oil immersion objective (Zeiss Microscopy GmbH, Germany). The software ZEN 2012 Black Edition (Zeiss Microscopy GmbH, Germany) and ImageJ 1.54f (RRID: SCR_003070) were used for analysis and quantification of the images. For each condition ROIs in the perinuclear and peripheral area were quantified in ZEN 2012. Furthermore, mitochondrial spread was determined by using the line tool across the major axis of the network, in ImageJ. The plot profile was taken as reference with a threshold value of 50 RFU to determine the borders of the network and quantify its diameter. Similarly, the nuclear gap was determined, yet instead of the outer edges the inner edges were taken as reference. For the 4 h incubation experiments the imaging was performed with a Lionheart FX Automated microscope from BioTek (Vermont, USA, RRID: SCR_019744), to allow for faster acquisition time, necessary due to the shorter kinetics of this experimental setup. Image acquisition and signal intensity quantification was performed using GEN5 Microplate Reader and Imager Software Version 3.05 from BioTek (Vermont, USA, RRID: SCR_017317). Mitochondrial area was determined via ImageJ.

#### Untargeted proteome analysis

##### Sample preparation

For proteome analysis, T24 cells were cultured in 6-well plates and treated for 24 h with 10 nM bafilomycin A1, 0.5 mM H_2_O_2_ or DMSO matched control medium. To obtain the nuclear proteins, first the supernatant was discarded, and adherent cells were washed twice with PBS. After complete removal of washing buffer, cells were lysed in isotonic lysis buffer (10 mM HEPES/NaOH, pH 7.4, 0.25 M sucrose, 10 mM NaCl, 3 mM MgCl_2_, 0.5 % Triton X-100, 1 mM PMSF, and 1 μg/ml each of pepstatin, leupeptin and aprotinin) by applying mechanical shear stress. After centrifugation at 2300 g and 4°C for 5 min, the supernatant containing the cytoplasmic proteins was removed and the pellets, containing the nuclear proteins, were incubated with 500 mM NaCl for 10 min before they were 1:10 diluted with NP-40 buffer for another 15 min. After centrifugation at 2300 g and 4°C for 5 min, the nuclear proteins were recovered in the supernatant and precipitated overnight with ice-cold ethanol at −20°C. Precipitated proteins of all samples were dissolved in sodium deoxycholate lysis buffer (SDC; 0.4 g SDC, 500 μL Tris·HCl (pH 8.8)).

Protein concentrations of nuclear extracts of T24 cells were determined via bicinchoninic acid (BCA) assay. For proteomic analyses, an adapted version of the EasyPhos workflow was applied as previously described.^157^^,^^158^ Briefly, 20 μg of protein was reduced and alkylated with 100 mM TCEP and 400 mM 2-CAM, respectively. Afterwards, enzymatic digestion of proteins was performed using a Trypsin/Lys-C mixture (1:100 Enzyme to Substrate ratio) at 37°C for 18 h. Thereafter, the peptide solution was desalted by first drying it to approximately 20 μL, mixing it with loading buffer containing 1% TFA in isopropanol and loading it on SDB-RPS StageTips. After two washing steps, peptides were eluted with 60% ACN and 0.005% ammonium hydroxide solution, dried and stored at −20°C until LC-MS analyses.

##### LC-MS/MS analysis

LC-MS/MS analysis was performed as described previously.^159^ Samples were analyzed as technical duplicates in data-dependent acquisition mode by label free quantification (LFQ) shotgun proteomics. In short, dried peptide samples were reconstituted in 5 μL formic acid (30%) containing four synthetic standard peptides at 10 fmol each and diluted with 40 μL loading solvent (98% H2O, 2% ACN, 0.05% TFA). Thereof, 5 μL were injected into the Dionex Ultimate 3000 nano high performance liquid chromatography (HPLC)-system (Thermo Fisher Scientific, RRID: SCR_019840). Pre-concentration of peptides was achieved on a pre-column (2 cm × 75 μm C18 Pepmap100; Thermo Fisher Scientific) run at a flow rate of 10 μL/min using mobile phase A (99.9% H2O, 0.1% FA). Subsequent peptide separation was performed on an analytical column (25 cm × 75 μm 25 cm Aurora Series emitter column (Ionopticks)) by applying a flow rate of 300 nL/min and using a gradient of 8%–40% mobile phase B (79.9% ACN, 20% H2O, 0.1% FA) over 95 min, resulting in a total LC run time of 135 min including washing and equilibration steps. Mass spectrometric analyses were performed using the timsTOF Pro mass spectrometer (Bruker) equipped with a captive spray ion source run at 1700 V. Further, the instrument was operated in the Parallel Accumulation-Serial Fragmentation (PASEF) mode. A scan range (m/z) from 100 to 1700 to record MS and MS/MS spectra and a 1/k0 scan range from 0.60–1.60 V.s/cm2 resulting in a ramp time of 100 ms to achieve trapped ion mobility separation were set as further parameters. All experiments were performed with 10 PASEF MS/MS scans per cycle leading to a total cycle time of 1.16 s.

##### LC-MS/MS data processing and evaluation

Identification as well as label-free quantification (LFQ) of proteins and statistical analyses were performed using publicly available software package MaxQuant 1.6.17.0 (RRID: SCR_014485) running the Andromeda search engine.^155^ Therefore, raw data were searched against the SwissProt database “homo sapiens” (version 141219 with 20380 entries). General search parameter included an allowed peptide tolerance of 20 ppm, a maximum of 2 missed cleavages, carbamidomethylation on cysteins as fixed modification as well as methionine oxidation and N-terminal protein acetylation as variable modification. A minimum of two peptide identifications per protein, at least one of them unique was set as search criterium for positive identifications. Furthermore, match between runs was performed using a match time window of 0.7 min and a match ion mobility window of 0.05 as well as a 20 min alignment time window and an alignment ion mobility of 1. For both peptides and proteins a false discovery rate (FDR) of less than 0.01 was applied.

LC-MS data evaluation as well as statistical analysis was accomplished using the Perseus software (version 1.6.14.0, RRID: SCR_015753).^160^ Therefore, identified proteins were filtered for reversed sequences as well as common contaminants and annotated according to the different treatment groups. LFQ values of technical duplicates were averaged and the biological replicates considered as independent. Prior to statistical analysis, LFQ intensity values were transformed (log2(x)), and proteins were additionally filtered for their percentage of independent identifications (70% in at least one group). Afterwards, missing values were replaced from a normal distribution (width: 0.3; down shift: 1.8). Two-sided t-tests as well as statistics for volcano plots were performed applying an FDR of 0.05 and a S0 (x-value of the hyperbolic tangent of the function separating significant events) of 0.1. The mass spectrometry proteomics data have been deposited to the ProteomeXchange Consortium via the PRIDE^154^ partner repository with the dataset identifier PXD060036.

#### DCF-DA assay

Cellular ROS production was quantified using, 2′,7′-dichlorofluorescin diacetate (DCF-DA).^161^ Cells were seeded in black 96-well plates and grown at 37°C for 48 h. The cells were treated for 4 h, bafilomycin (0.1-1-10 nM), catalase (100 U/mL) and the combination of the two, as well as H_2_O_2_ (0.5 mM) as positive control. After washing with live cell imaging solution (LCIS) cells were incubated with DCF-DA solution (50 μM; 100 μL per well) for 15 min. Afterwards, the cells were washed again with LCIS. Measurements were performed using a Synergy H1 plate reader (BioTek, Bad Friedrichshall, Germany, RRID: SCR_019748) at excitation and emission wavelengths of 485 nm and 530 nm.

#### Immunofluorescence

For immunofluorescence experiments previously used protocols were slightly adapted.^23^^,^^95^^,^^162^ Cells were seeded in μ-Slide 8 Well ibiTreat sildes (REF: 80.826, Ibidi GmbH, Gräfelfing, Germany) and allowed to grow for 48 h, afterwards the cells were treated for 4 h, with bafilomycin (10 nM), catalase (100 U/mL) and the combination of the two. Fixation was performed using 3.5% formaldehyde solution. Cells were washed with PBS and formaldehyde was quenched using a glycine PBS solution. Following another PBS washing step cells were permeabilized with 0.2% Triton X-100 for 15 minutes and non-specific sites were blocked with 2% donkey serum in PBS. Primary antibodies (anti-Lamin A/C Ref: sc-376248, RRID: AB_10991536, anti-Phosphoserine/threonine/tyrosine Ref: 61-8300, RRID: AB_2533941) were incubated for 2 h at 4°C. Cells were washed 3 times with washing buffer (0.05% Triton-X in PBS) and 2 times with PBS. For the Lamin A/C and actin staining secondary antibody (Alexa Fluor 647 anti-mouse A31571, Lot: 2260928, RRID: AB_162542) and phalloidin (Invitrogen Oregon Green® 488) and for the Lamin A/C and phosphorylation staining other secondary antibodies (Alexa Fluor 488 donkey anti-mouse A21202 and Alexa Fluor 647 donkey anti-rabbit A31573) were incubated overnight and the same washing steps were repeated. Finally, cells were covered with mounting medium containing DAPI (ROTH Roti®Mount FluorCare DAPI ArtNr: HP 20.1) to stain cell nuclei. Imaging was performed with a Lionheart FX Automated microscope from BioTek (Vermont, USA, RRID: SCR_019744). Image acquisition and quantification was performed using GEN5 Microplate Reader and Imager Software Version 3.05 from BioTek (Vermont, USA, RRID: SCR_017317). Morphology of Lamin A/C was evaluated using ImageJ 1.54f (RRID: SCR_003070). The imaging of the combined Phosphorylation and Lamin A/C staining was performed with using a Zeiss LSM710 laser scanning confocal microscope (ELYRA PS.1 system, RRID: SCR_018063) with a 63X/1.46 Plan-Apochromat oil immersion objective (Zeiss Microscopy GmbH, Germany). The software ZEN 2012 Black Edition (Zeiss Microscopy GmbH, Germany) and ImageJ 1.54f were used for analysis and quantification of the images. Cells were quantified using the Lamin A/C mask for reference and measuring the intensity of the Lamin A/C and phosphorylation signal. Secondly, colocalization of the phosphorylation and Lamin A/C signal was investigated, firstly in the total image and then in ROIs selected in center of the lamina mask as well as the border.

#### γH2AX and nuclear morphology

Cells were seeded in μ-Slide 8 Well ibiTreat sildes (REF: 80.826, Ibidi GmbH, Gräfelfing, Germany) and treated for 4 h with bafilomycin (10 nM), catalase (100 U/mL) and the combination of the two.^95^ Cells were fixed with 100 μL ice-cold methanol for 20 minutes. Afterwards 3 washing steps with PBS-T (PBS with 0.1% Tween-20) were performed. Unspecific sites were blocked with 1% BSA in PBS-T for 1 h. Afterwards primary antibody (Anti-phospho-Histone H2A.X (Ser139) mouse Antibody, 1:500) was incubated for 2 h. Cells were washed trice with PBS-T and incubated with secondary antibody solution (Alexa Fluor 647 anti-mouse A31571, Lot: 2260928, 1:400) overnight. After three PBS-T washing steps cells were embedded in DAPI containing mounting medium (ab104139, Abcam) and imaged using a Lionheart FX automated microscope (BioTek Instruments Inc., Vermont, USA, RRID: SCR_019744) using the GEN5 microplate and Imager software version 3.05. for imaging and quantification (BioTek Instruments Inc., Vermont, USA, RRID: SCR_017317). For γH2AX the mean intensity per cell was measured and the mean per image was taken per optical field. Furthermore, the shape descriptors (Circularity, roundness, solidity, aspect ratio and area)^40^ of the DAPI signal (i.e., nuclear morphology) were quantified using ImageJ 1.54f (RRID: SCR_003070).

#### Atomic force microscopy (AFM)

For the investigation of cell stiffness, a JPK NanoWizard® 4 XP atomic force microscope (Bruker, Germany) coupled with an inverted Olympus IX73 optical microscope was used.^52^^,^^163^^,^^164^ Cells were seeded in tissue culture dishes (REF: 40 93040, TPP Techno Plastic Products AG, Switzerland). After this, cells were incubated with the respective treatments for 4 h. After a LCIS (pre-warmed to 37°C) washing step, cells were measured in LCIS using QI™ mode and PFQNM-LC-A-CAL (Bruker, Figures 4, 6, S3, and S8), or PFQNM-LC-V2 AFM tips (Bruker, Figure 3), with calibrated spring constants k ranging from 0.070 N/m to 0.090 N/m. QI™ settings used are the following: Z-length: 1000 nm; applied force 0.2 nN; speed 100 μm/s. Using the optical image as reference viable cells were selected and imaged in 25 μm × 25 μm areas. Imaging time per dish was limited to 1 h to ensure stable cell viability. *N* ≥ 10 cells were measured per condition from at least three independent cell preparations (biological replicates).

Individual force curves were processed to obtain data of the Young’s modulus. For this, areas of 3.1 μm × 3.1 μm were selected aligning with the nuclear, perinuclear and cytoplasmic regions visible in the overlay with optical image. Raw data was processed using JPK NanoWizard® Data processing software. A representative force curve of the cell was chosen to setup the calculation of the Young’s modulus. To start, the average value of a section in the baseline was subtracted from the whole curve, to remove the baseline offset in vertical deflection. Following this, vertical deflection was plotted against the vertical tip position. The reference force height was determined at the height value at 50% of the applied setpoint force. To calculate the Young’s modulus the elasticity fit model was used.^165^^,^^166^ The Hertz/Sneddon fit was calculated choosing a paraboloid tip shape with a 70 nm tip radius and a Poisson’s ratio value of 0.50. Force curves were batch-analyzed in the areas of the nuclear, perinuclear, and cytoplasmic regions and median value of the Young’s modulus was calculated.^167^^,^^168^

#### Seahorse assay

The seahorse assay to measure mitochondrial function was performed according to the manufactures instructions and previously published literature.^105^^,^^169^ Cells were seeded in Seahorse XF cartridges using standard cultivation medium at densities which resulted in a basal respiration rate of around 70 pmol/min (optimal range as per supplier 20–150 pmol/mL). Two wells were incubated with cell free medium, to act as blanks. Cells were rested for 1 h at room temperature to allow attachment and then incubated for 24 h (37°C, 5% CO_2_). On the same day the sensor cartridge was hydrated with sterile H_2_O (200 μL/Well and 400 μL/moat) and incubated overnight at 37°C in a CO_2_ free incubator. An aliquot of XF calibrant was incubated in the same manner. Similarly, the assay medium was prepared (9.7 mL Seahorse XF DMEM + 0.1 mL SH-Glucose + 0.1 mL SH-Pyruvate + 0.1 mL SH-Glutamine). On the next day the cell plate was incubated with the pretreatment (BAFI 10 nM, 4 h). The water was removed, and the sensors were incubated for 1 h in XF calibrant before the assay. The sensor cartridge was further loaded with the respective drugs in order of application to result in concentrations on the cells of (1. 0.5 μM Oligomycin, 2. 1.5 μM CCCP and 3. 0.5 μM Antimycin A and 0.5 μM Rotenone). To start 3 mixing and measurement circles were performed to measure basal respiration, following this the first drug was injected followed by a second circle of 3 mixing and measurements, and repeated in the same manner for the other treatments. The raw data of the respiratory rates was evaluated in OriginPro. Determined were the basal respiration (average of the first three measurement points minus the average of the non-mitochondrial respiration (after AA/ROT) as well as the maximum respiration (average after CCCP minus non-mitochondrial respiration).

#### Alkaline comet assay

In order to assess genotoxicity, the comet assay was performed in its alkaline form.^95^^,^^96^ Cells were seeded in 3.5 cm petri dishes and grown for 48 h. Cells were incubated for 4 h with bafilomycin (10 nM), catalase (100 U/mL), or the combination of the two. Object slides were prepared by applying a layer of normal melting agarose (0.8% NMA in phosphate buffer: 137 mM NaCl, 2.7 mM KCl, 1.5 mM KH_2_PO_4_, 8.1 mM Na_2_PO_4_). On top gel patches of NMA were formed by pipetting 65 μL 0.8% NMA and covering with a lid and cooling the slides on ice. In the final minutes of the incubation time a positive control was prepared using UV radiation for 1 min (31.2 J). After two PBS washes cells were detached using 250 μL trypsin. 500 μL of complete medium was added and 30000 cells were centrifuged at 4°C and 420 rcf for 10 min. Cell pellets were resuspended in low melting agarose (0.8% LMA, 37°C) and pipetted onto the gel patches and covered with a small slide. After cooling for 10 min slides were put into a Coplin beaker and lysed overnight in a buffer containing DMSO, Triton X-100, and N-lauryl sarcosine. For formamidopyrimidine DNA glycosylase (FPG) treatment, cells were washed three times with FPG buffer (40 mM HEPES, 0.1 M KCl, 0.5 mM EDTA, 0.2 mg/mL BSA) and treated with either 50 μL FPG buffer with or without the FPG enzyme. Cells were incubated for 30 min at 37°C, before beginning the electrophoresis.

Slides were allowed to equilibrate in an alkaline buffer (60 mL NaOH, 10 mL 200 mM EDTA filled to 2 L) for 20 min and electrophoresis was performed in the same buffer for 20 min at 25 V and 300 mA in the dark. Afterward slides were washed with a neutralizing buffer (0.4 M tris/HCl, pH 7.5) and stained with ethidium bromide solution (20 μg/mL, 35 μL per patch). Imaging and quantification were performed using the Comet Assay IV software. In total 50 cells per patch were measured and the mean was calculated. In total, *n* = 6 patches taken from three biological replicates were evaluated.

### Quantification and statistical analysis

Statistical calculations were performed in OriginPro2023b (RRID: SCR_014212) or Perseus 1.6 (Proteome Data, RRID: SCR_015753), One-Way ANOVA was used in concentration dependent data sets, Mann-Whitney for direct comparison of datasets with datapoints *n* < 23 and Student’s *t*-Test for all other data sets. In the graphs, whiskers represent standard deviation (SD), the boxes 25–75%, the median is depicted as a line and the mean as a square. Statistical details of the experiments (i.e., number of quantified cells, images or ROIs) and the statistical test used can be found in the figure legends corresponding to the individual data sets.
