# Supplementary material for: Retrograde rearrangement of mitochondria correlates with nuclear deformation and genotoxic damage
Source: iScience. 2025 Jun 19;28(8):112955. doi: 10.1016/j.isci.2025.112955 (PMC12302250; doi:10.1016/j.isci.2025.112955)
Supplement: Document S1. Figures S1–S11 [file mmc1.pdf]

iScience, Volume *28*

Supplemental information

Retrograde rearrangement of mitochondria correlates with nuclear deformation and genotoxic damage

Maximilian Jobst, Francesco Crudo, Doris Marko, Andrea Bileck, Samuel Matthias Meier-Menches, Christopher Gerner, and Giorgia Del Favero

Supplementary Figure 1: Additional proteome signature related to phosphorylation in T24 cells treated with bafilomycin, related to Figure 1

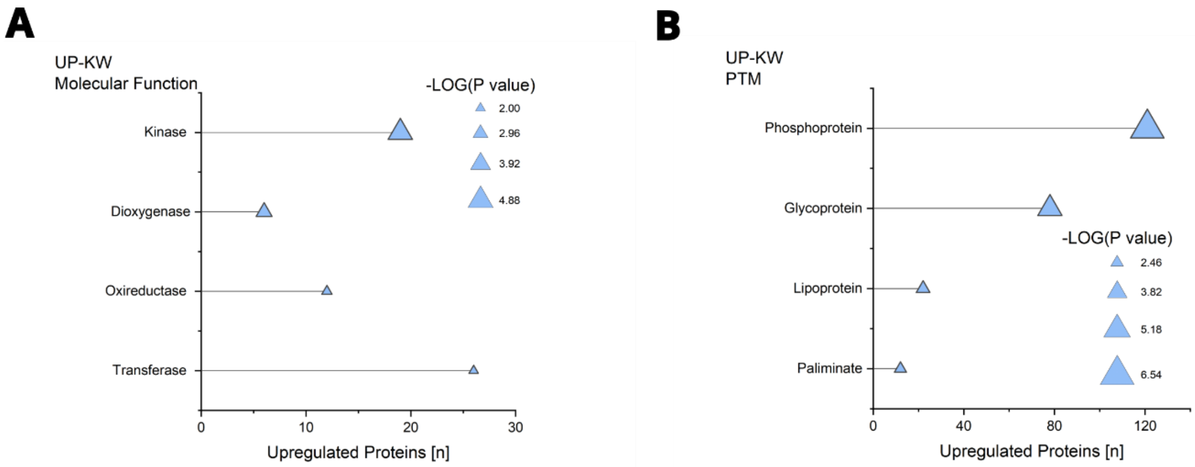

(A) Upregulated pathways according to DAVID bioinformatics analysis, using uniprot keywords for the category molecular function in cells treated with bafilomycin 10 nM for 24 h.

(B) Upregulated pathways according to DAVID bioinformatics analysis, using uniprot keywords for the category post translational modifications (PTM). (Figure 1A and 1B) Depicted is the number of upregulated proteins, the size of the symbol (triangle) indicated the statistical significance -Log (p-value).

Supplementary Figure 2: T24 mitochondrial signal intensity is unaltered by bafilomycin, related to Figure 2

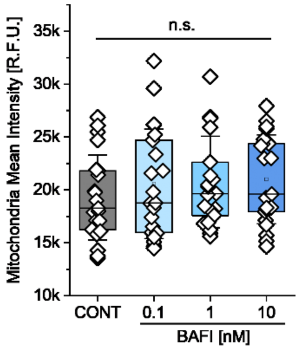

Mitochondrial signal intensity of T24 cells treated with bafilomycin, catalase and the combination of the two for 24 h. Intensity was measured for individual cells and averages per optical field were formed (N ≥ 20 optical fields). Results are shown as boxplots, whiskers represent SD and boxes represent the range from 25 to 75 percentage. Statistical significance was determined using one-way ANOVA test with Fisher LSD test for means comparison (n.s. $p > 0.05$).

Supplementary Figure 3: T24 Cell area is unaltered by bafilomycin and catalase, related to Figure 4

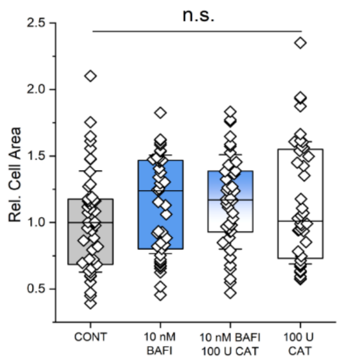

Relative cell area of T24 cells treated with bafilomycin, catalase and the combination of the two for 4 h. Results are shown as boxplots, whiskers represent SD and boxes represent the range from 25 to 75 percentage. Statistical significance was determined using Student's *t*-test for means comparison (n.s. $p > 0.05$).

Supplementary Figure 4: Additional AFM maps of T24 cells treated with bafilomycin and catalase, related to Figure 4

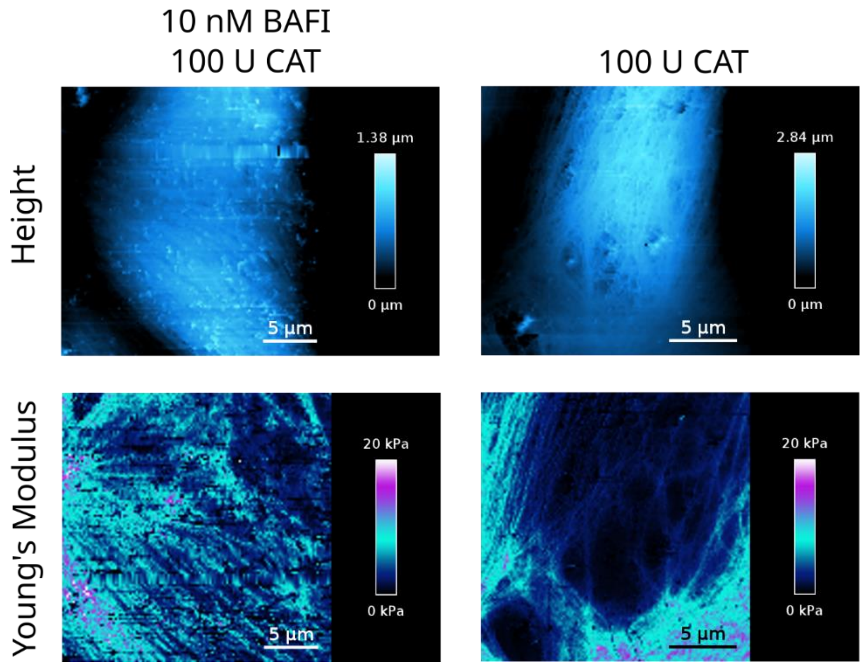

Additional representative height and Young's modulus maps of T24 cells, cells treated with 10 nM bafilomycin and catalase in combination and 100 U/ml catalase alone for 4h.

Supplementary Figure 5: Additional morphological parameters of T24 nuclei of cells treated with bafilomycin and catalase, related to Figure 5

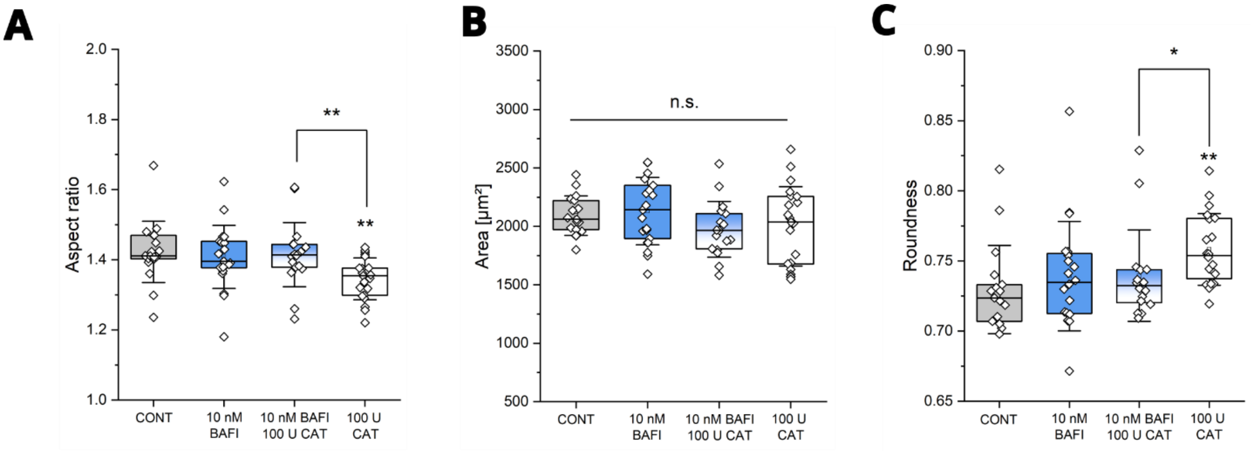

(A-C) Morphological parameters; Aspect Ratio (A), Area (B) and Roundness (C) in T24 cells treated with bafilomycin, catalase and the combination of the two for 4 h. (A-C) Results are shown as boxplots, whiskers represent SD and boxes represent the range from 25 to 75 percentage. Statistical significance was determined using one-way ANOVA test with Fisher LSD test for means comparison (n.s. $p > 0.05$, * < 0.05, ** < 0.01).

Supplementary Figure 6: Actin signal remains unchanged following bafilomycin and catalase incubation in T24 cells, related to Figure 5

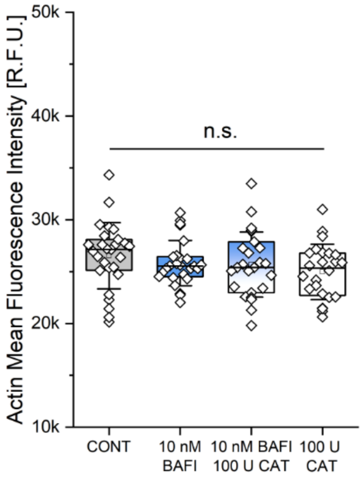

Quantification of the actin staining in T24 bladder cancer cells treated with bafilomycin, catalase, and the combination of the two. Results are shown as boxplots, whiskers represent SD and boxes represent the range from 25 to 75 percentage. Statistical significance was determined using one-way ANOVA test with Fisher LSD test for means comparison (* $p < 0.05$, ** $p < 0.01$, *** $p < 0.001$).

Supplementary Figure 7: Comparison of the comet assay with and without FPG treatment, related to Figure 5

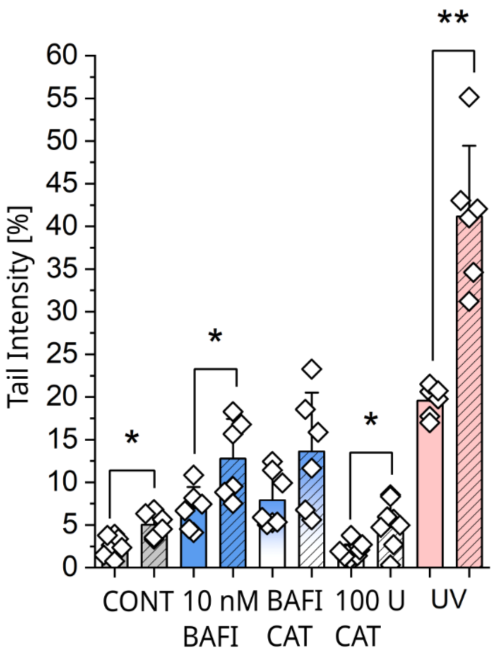

Additional statistical comparison of the comet assays described in Figure 5E and 5F of the main manuscript. Shown here are the p-Values calculated per Mann-Whitney test between the same conditions with and without FPG treatment (* $p < 0.05$, ** $p < 0.01$).

Supplementary Figure 8: Lysosomal signal responds similarly in T24 cells, SK-OV-3 and HCT 116 treated with 10 nM bafilomycin, related to Figure 7

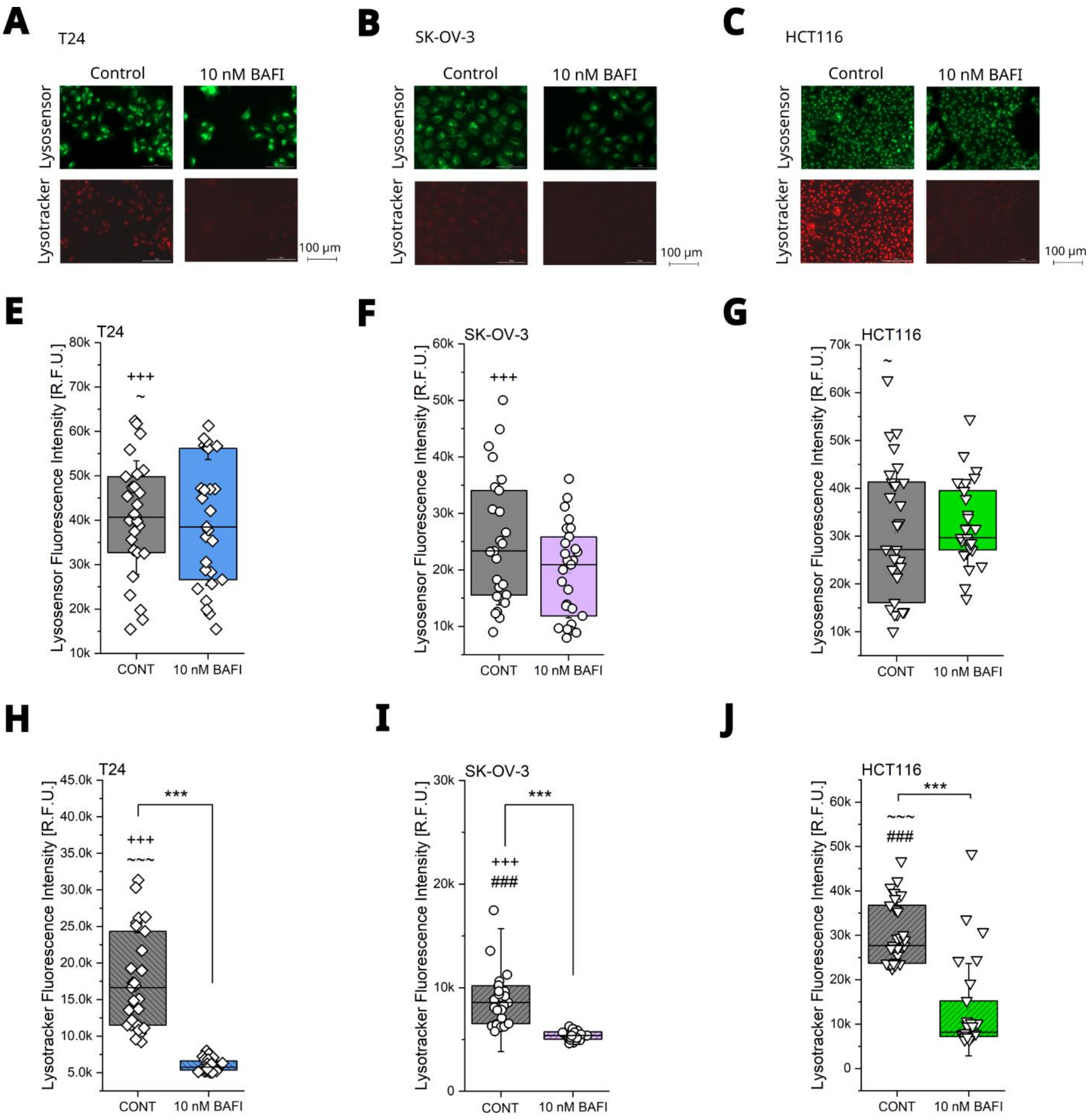

(A,B,C) Representative images of lysosomal staining using LysoTracker™ and LysoSensor™ in T24 (A), SK-OV-3 (B), HCT116 (C) cells controls and 10 nM bafilomycin treatment (4 h). Scale bar 100 µm.

(E,F,G) Quantification of the LysoSensor™ imaging in in T24 (E), SK-OV-3 (F), HCT116 (G) cells. At least N ≥ 27 cells were quantified per condition (3 biological replicates)

(H,I,J) Quantification of the LysoTracker™ imaging in T24 (H), SK-OV-3 (I), HCT116 (J) cells. At least N ≥ 27 cells were quantified per condition (3 biological replicates). Statistical significance was determined via Student's *t*-test (~ $p < 0.05$; ***,+++,~~~,### $p < 0.001$) * indicates differences among the same cell line. + indicates differences between T24 and SK-OV-3, # between SK-OV-3 and HCT116 and ~ between T24 and HCT116 control cells.

Supplementary Figure 9: Comparisons of cell stiffness of T24, SK-OV-3, and HCT116 cells, related to Figure 7

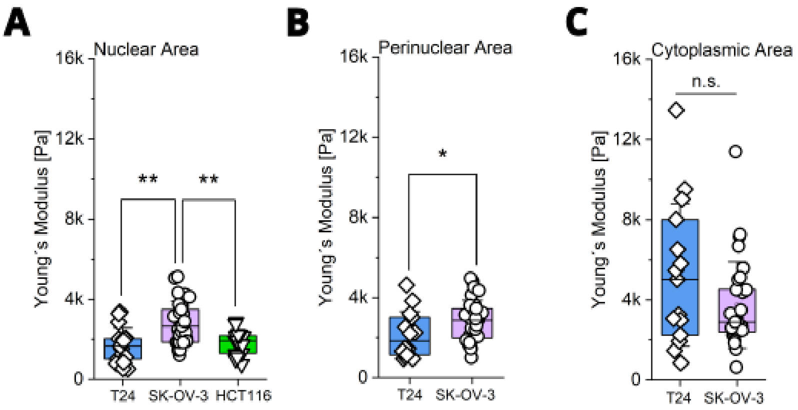

(A-C) Comparison of the Young's modulus in the nuclear (A), perinuclear (B) and cytoplasmic area (C) of T24, SK-OV-3 and HCT 116 control cells (Taken from the experiments depicted in Figure 4 and 7 respectively). Results are shown as boxplots, whiskers represent SD and boxes represent the range from 25 to 75 percentage. For the statistical evaluation of the results Mann-Whitney test was performed (* $p < 0.05$, ** $p < 0.01$). N ≥ 10 cells were quantified per condition (N ≥ 3 biological replicates).

Supplementary Figure 10: Nuclear morphology in SK-OV-3 and HCT 116 cells following bafilomycin and catalase incubation in T24 cells, related to Figure 7

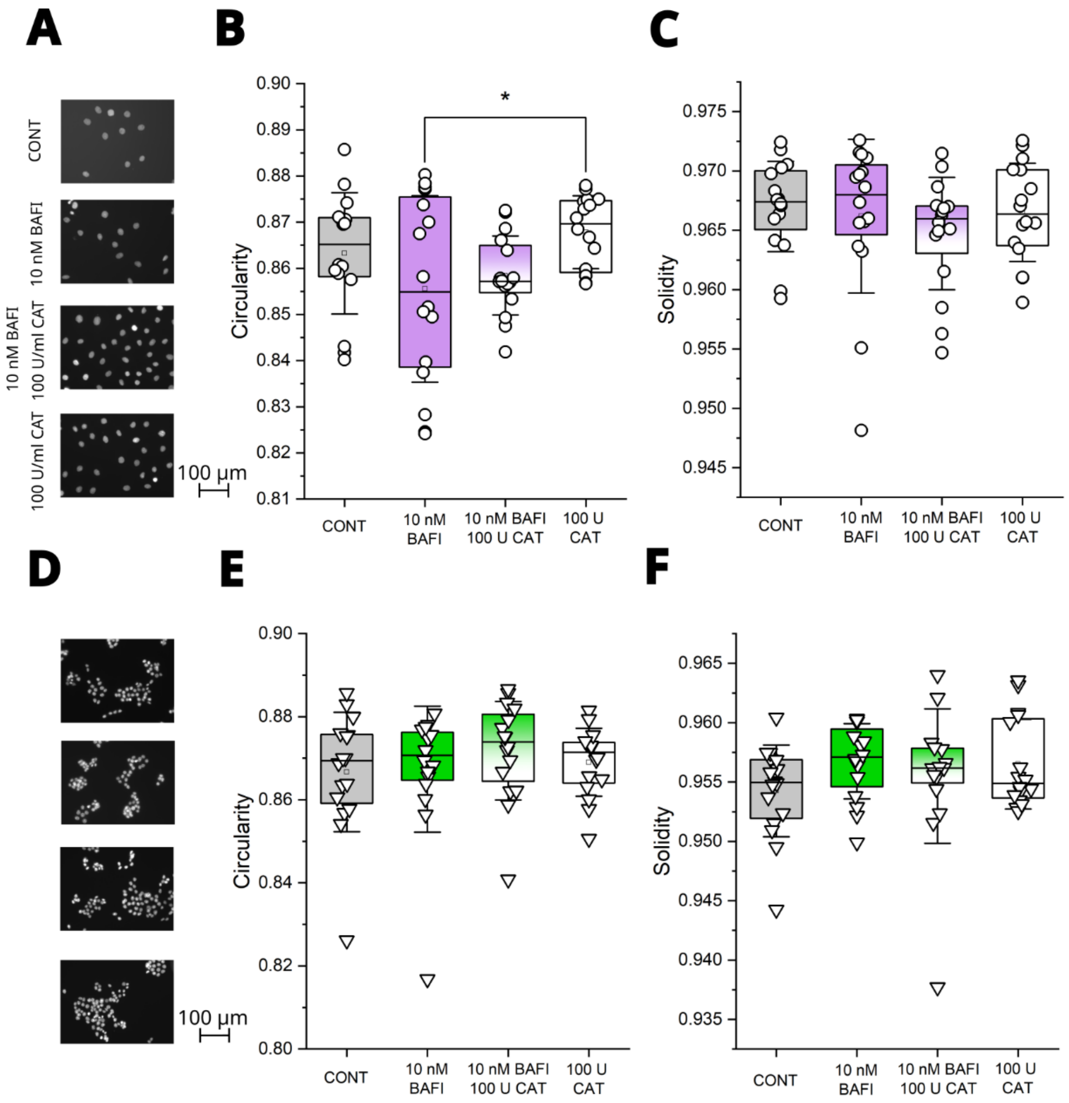

(A-D) Morphological analysis of cell nuclei stained with DAPI, in SK-OV-3 (A,B,C) and HCT 116 (D,E,F). Representative images of SK-OV-3 (A) and HCT116 (E). Shown are circularity (B,C) and solidity (E,F). Cells were treated with 10 nM bafilomycin, 100 U/mL catalase and the combination of the two for 4 h. Results are shown as boxplots, whiskers represent SD and boxes represent the range from 25 to 75 percentage. Statistical significance was determined using one-way ANOVA test with Fisher LSD test for means comparison (* $p < 0.05$, ** $p < 0.01$, *** $p < 0.001$). Scale bar 100 µm.

Supplementary Figure 11: Colocalization of Lamin A/C and phosphorylation in T24 cell treated with bafilomycin 1 and 10 nM in the full optical field, related to Figure 9

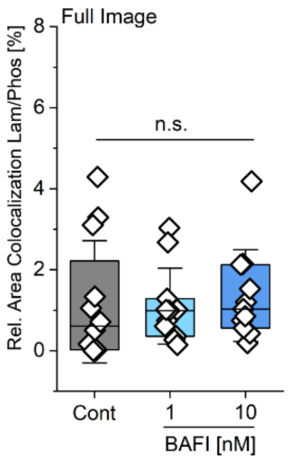

Colocalization of Lamin A/C and phosphorylation signal calculated for full optical field. T24 cells treated with 1 and 10 nM bafilomycin and control cells. N = 12 optical fields. Results are shown as boxplots, whiskers represent SD and boxes represent the range from 25 to 75 percentage. For the statistical evaluation of the results one-way ANOVA-tests were performed (n.s. $p > 0.05$).
